# TFEB activation triggers pexophagy for functional adaptation during oxidative stress under calcium deficient-conditions

**DOI:** 10.1186/s12964-024-01524-x

**Published:** 2024-02-21

**Authors:** Laxman Manandhar, Raghbendra Kumar Dutta, Pradeep Devkota, Arun Chhetri, Xiaofan Wei, Channy Park, Hyug Moo Kwon, Raekil Park

**Affiliations:** 1 Department of Biomedical Science and Engineering, Gwangju Institute of Science and Technology, Gwangju, 61005 Republic of Korea; 2 School of Life Sciences, Ulsan National Institute of Science and Technology, Ulsan, Republic of Korea; 3 Present address: Department of Chemistry (Biochemistry Division) Crosley Tower, University of Cincinnati, Cincinnati, Ohio 45221 USA

**Keywords:** Calcium, Peroxisome, Autophagy, Catalase, ROS, TFEB

## Abstract

**Background:**

Calcium is a ubiquitous intracellular messenger that regulates the expression of various genes involved in cell proliferation, differentiation, and motility. The involvement of calcium in diverse metabolic pathways has been suggested. However, the effect of calcium in peroxisomes, which are involved in fatty acid oxidation and scavenges the result reactive oxygen species (ROS), remains elusive. In addition, impaired peroxisomal ROS inhibit the mammalian target of rapamycin complex 1 (mTORC1) and promote autophagy. Under stress, autophagy serves as a protective mechanism to avoid cell death. In response to oxidative stress, lysosomal calcium mediates transcription factor EB (TFEB) activation. However, the impact of calcium on peroxisome function and the mechanisms governing cellular homeostasis to prevent diseases caused by calcium deficiency are currently unknown.

**Methods:**

To investigate the significance of calcium in peroxisomes and their roles in preserving cellular homeostasis, we established an in-vitro scenario of calcium depletion.

**Results:**

This study demonstrated that calcium deficiency reduces catalase activity, resulting in increased ROS accumulation in peroxisomes. This, in turn, inhibits mTORC1 and induces pexophagy through TFEB activation. However, treatment with the antioxidant N-acetyl-l-cysteine (NAC) and the autophagy inhibitor chloroquine impeded the nuclear translocation of TFEB and attenuated peroxisome degradation.

**Conclusions:**

Collectively, our study revealed that ROS-mediated TFEB activation triggers pexophagy during calcium deficiency, primarily because of attenuated catalase activity. We posit that calcium plays a significant role in the proper functioning of peroxisomes, critical for fatty-acid oxidation and ROS scavenging in maintaining cellular homeostasis. These findings have important implications for signaling mechanisms in various pathologies, including Zellweger’s syndrome and ageing.

**Supplementary Information:**

The online version contains supplementary material available at 10.1186/s12964-024-01524-x.

## Background

Calcium (Ca^2+^) functions as a common secondary messenger in all eukaryotes, and variations in its cytoplasmic or organelle-specific content have been observed in various biological processes. Calcium signaling can be initiated by either calcium influx through the plasma membrane or calcium efflux from the endoplasmic reticulum (ER). The ER can release calcium into the cytosol or to adjacent organelles via specific compartments and membrane contact sites [1].

The transportation of calcium to subcellular organelles underscores its crucial role in cellular signaling. Numerous studies have delved into the significance of calcium in generating reactive oxygen species (ROS) in mitochondria and have demonstrated that ROS can trigger the release of calcium from the lysosome into the cytosol from the lysosome [2, 3].

These studies also offer evidence of calcium storage in subcellular organelles like the ER, mitochondria, lysosome, and peroxisome [4]. Likewise, the calcium concentration also varies in cellular organelles like ER: 100-800uM, mitochondria: 100 nM-800uM, lysosome: 500uM, cytosol: 100-200 nM and peroxisome: 600 nM. However, an in-depth exploration of calcium’s function in these organelles, particularly peroxisomes, remains elusive.

Peroxisomes are prevalent eukaryotic organelles that play a variety of roles in metabolic processes and are particularly abundant in liver tissue. Their functions include the α-oxidation of phytanic acid and the β-oxidation of very long-chain fatty acids (VLCFA). ROS are byproducts of oxidative reactions, with hydrogen peroxide (H_2_O_2_) being produced by various oxidases, including acyl-CoA oxidases, urate oxidases, and xanthine oxidases [5]. Peroxisomes also house several anti-oxidant enzymes, such as glutathione peroxidase, superoxide dismutase (SOD), and catalase [6].

Autophagy is an evolutionarily conserved degradation and recycling process that relies on lysosomal hydrolases and acid lipases. It operates alongside proteasomal degradation, mediating the turn-over of damaged, excess, or unwanted cellular constituents, including long-lived proteins, protein aggregates, lipids, and complete organelles, like mitochondria and peroxisomes [7]. Autophagy can mediate the non-selective degradation of cytoplasmic components as well as the selective degradation of cellular components including lipids (lipophagy) [8]; the ER (ER-phagy) [9]; mitochondria (mitophagy) [10]; and peroxisomes (pexophagy) [11].

Calcium has been connected to mammalian target of rapamycin (mTOR) and AMPK, two autophagic signaling pathways. Furthermore, there is substantial evidence supporting the roles of Ca^2+^ signals, Ca^2+^ channels, and Ca^2+^ sources in regulating autophagic processes [12].

The mTOR signaling system adapts to various stimuli, synchronizing energy, growth signals, and nutritional abundance with cell growth and division [13]. Dysregulation of the mTOR complex 1 (mTORC1) signaling pathway is implicated in conditions such as diabetes, metabolic disorders, and cancer [14]. In nutrient-rich environments, mTORC1 is activated, directly phosphorylating and inhibiting autophagy-related proteins. Conversely, low-nutrient levels lead to mTORC1 inactivation, initiating autophagy by disassociating from the ULK complex. Recently, transcription factor EB (TFEB), a member of the basic helix-loop-helix-zipper family, was discovered to regulate genes involved in lysosomal biogenesis and autophagy [15, 16]. In certain scenarios like starvation or lysosomal malfunction, mTOR-mediated phosphorylation controls the activity and subcellular location of TFEB [17]. Metabolic abnormalities, including increased food intake, insulin resistance, hepatic steatosis, hyperinsulinemia, and muscular atrophy, result from defective autophagy [18, 19]. Conversely, restoration of TFEB in the livers of obese mice, along with enhanced autophagy activity, mitigates obesity-induced insulin resistance [20, 21]. Several preclinical studies focusing on autophagy in various liver disorders are underway [22].

While previous research has highlighted the vital role of calcium in cellular signaling and its involvement with various organelles, there remains a significant gap in our understanding of how calcium impacts peroxisome function, particularly in relation to cellular homeostasis. Thus, this study aimed to address this gap by investigating the importance of calcium levels in peroxisome function. This study demonstrated that calcium depletion affects peroxisome prior than others indicating peroxisomal calcium for calcium homeostasis in cell. Also, indicates the presence of calcium channels in peroxisomes.

## Materials and methods

### Cell culture

Standard cell culture methods were used. AML12 and mouse embryonic fibroblast (MEF) cells were maintained in Dulbecco’s modified Eagle medium (DMEM, Gibco-BRL, Grand Island, NY, USA) supplemented with 10% fetal bovine serum (FBS, Gibco-BRL, Grand Island, NY, USA), 100 IU/mL penicillin (Invitrogen, Carlsbad, CA, USA), and 100 μg/mL streptomycin (Invitrogen, Carlsbad, CA, USA) at 37 °C and 5% CO_2_ in humidified air.

### Calcium-deficient condition and chemical treatments

To prepare the calcium-deficient condition, cells were incubated in calcium-deficient medium (DMEM, Gibco-BRL, Grand Island, NY, USA) supplemented with 10% FBS, 100 IU/mL penicillin, and 100 μg/mL streptomycin. After 24 h, medium were replaced by calcium deficient medium (DMEM, Gibco-BRL, Grand Island, NY, USA) supplemented with 10% FBS, 100 IU/mL penicillin, and 100 μg/mL streptomycin for particular time to prepare respective duration of calcium-free conditions and harvested. For rescue experiments, cells were incubated in normal medium for 24 h and were replaced by calcium-deficient medium to incubate for 12 h, then switched to normal medium and harvested after an additional 12 h. Chloroquine (#C6628, Sigma-Aldrich, St. Louis, MO, USA) or *N*-Acetyl-L-cysteine (NAC, #A7250, Sigma-Aldrich, St. Louis, MO, USA) were added during calcium-deficient medium replacement.

### Western blot analysis

Cells were harvested and centrifuged at 1000 × g for 5 min at 4 °C. They were then homogenized in RIPA buffer (10 mM Tris-HCl [pH 7.6], 150 mM NaCl, 1% Triton X-100, 1% sodium deoxycholate, and 1 mM EDTA) mixed with 1 × of protease and phosphatase inhibitor cocktail (GenDEPOT). After centrifugation at 14,000 rpm for 10 min at 4 °C, the supernatant was collected. Protein concentration was measured, and lysates were boiled for 10 min at 97 °C, before immunoblotting with antibodies against catalase (#ab217793, Abcam, Cambridge, MA, USA), Pex14 (#ab183885, Abcam, Cambridge, MA, USA and #A303-085A, Bethyl Laboratories, Montgomery, TX, USA), PMP70 (#ab3421, Abcam, Cambridge, MA, USA), ACOX1 (#10957-1-AP, Proteintech, Rosemont, IL 6008, USA), Pex3 (#ab247042, Abcam, Cambridge, MA, USA), Pex1 (#13669-1-AP, Proteintech, Rosemont, IL 6008, USA), Pex19 (#14,713-1-AP, Proteintech, Rosemont, IL 6008, USA), Pex11β (#ab211508, Abcam, Cambridge, MA, USA), LC3 (#L8918, Sigma-Aldrich, St. Louis, MO, USA), SQSTM1/p62 (#H00008878-M01, Abnova, Taipei, Taiwan), ATG5 (#12994, Cell Signaling, Danvers, Massachusetts, USA), p-TFEB (#E9S8N, Cell Signaling, Danvers, Massachusetts, USA), TFEB (# ab264421, Abcam, Cambridge, MA, USA), α-tubulin (#2125, Cell Signaling, Danvers, Massachusetts, USA), CREB (#9197, Cell Signaling, Danvers, Massachusetts, USA), NBR1 (#16004-1-AP, Proteintech, Rosemont, IL 6008, USA) and β-actin (#sc-47778, Santa Cruz Biotechnology, Dallas, TX, USA).

### Cell fractionation

Cell pellet was washed with ice-cold PBS, scraped, and centrifuged at 1000 × *g* for 5 min at 4 °C. Next, the cell pellet was homogenized in 400 µL of buffer A (10 mM HEPES [pH 7.9], 50 mM NaCl, 0.1 mM EDTA, 1 mM DTT, and protease/phosphatase inhibitors cocktail). The homogenates were centrifuged at 1000 × *g* for 10 min at 4 °C, and the supernatant was transferred to a new tube. The supernatant was then centrifuged at 16,000 × *g* for 30 min at 4 °C. After centrifugation, the supernatant (cytosolic extract) was transferred to a new tube for protein-concentration measurement. The pellet from this process was re-suspended in 100 µL of buffer B (20 mM HEPES [pH 7.9], 0.4 M NaCl, 1 mM EDTA, 1 mM DTT, 10% glycerol (vol/vol), and protease/phosphatase inhibitors cocktail) and rotated at 4 °C for 1 h. After centrifugation at 16,000 × *g* for 30 min at 4 °C, the supernatant (nuclear extracts) was transferred to a new tube for protein measurement. After measuring the protein concentration, they were used for western blot analysis [23, 24].

### Immunofluorescence

Cells were fixed in 4% paraformaldehyde (PFA, Sigma Aldrich; HT5014) in PBS for 30 min at room temperature after being rinsed with PBS at a confluence of 50% to 70%. Following this, they were being washed three times with PBS and incubated in 0.5% triton X-100 in PBS. The cells were blocked in 3% bovine serum albumin for 1 h before being treated at 4 °C with 1:500 dilutions of anti-PMP70, anti-NBR1, and anti-LAMP1 antibodies for overnight. The cells were washed with PBS, stained for 1 h with fluorescent Alexa Fluor-488 and Fluor-568, washed twice with PBS, and then incubated at room temperature for 10 min with 10 mM 4′,6-diamidino-2-phenylindole (DAPI) in PBS. Cells were examined using either an IX71 fluorescence microscope (Olympus, Tokyo, Japan) or an Olympus FV1000 confocal laser scanning microscope after the coverslips had been mounted.

The co-localization of PMP70 and LAMP1 was measured using ImageJ software based on Mander’s overlap coefficient (OC) of co-localization, which varies from zero to one. A value of zero corresponds to non-overlapping images whereas a value of one reflects 100% co-localized between the images being analyzed.

### Pexophagy assay

To detect pexophagy, RPE1 cells expressing the monomeric red fluorescent protein (mRFP) –green fluorescent protein (GFP)-serine-lysine-leucine (SKL) plasmid were used [25, 26]. Cells were switched to calcium-deficient media for the specified amount of time after achieving 70% confluence. They were then fixed with 4% PFA for 30 min at room temperature after being rinsed with PBS. The coverslips were mounted, and a fluorescence microscope was used for examination. The percentage was calculated based on the number of cells displaying mRFP-labelled autolysosomes, indicating lysosomal transport, along with the peroxisomal reporter mRFP-EGFP-SKL.

### ROS measurement

ROS levels were assessed using Cell ROX deep red reagent (#C10422, Thermo-Scientific, Waltham, Massachusetts, USA). Cells were seeded in a 12-well plate and transferred to calcium-deficient media treated with NAC at 60% confluency. After the allotted time, the Cell ROX reagent was applied to the cells and incubated for 30 min at 37 °C at a final concentration of 5 µM. Cells were then washed with PBS and observed under a fluorescence microscope [27].

To measure the levels of mitochondria-specific ROS, Mito SOX red (#M36008, Thermo-Scientific, Waltham, Massachusetts, USA). Cells were seeded in a 12-well plate and transferred to calcium-deficient for particular time period. The final concentration of 1 uM Mito SOX were added and incubated for 30 min at 37 °C followed by three times washed with PBS. Two drops of NucBlue Live Cell Stain (#R37605, Thermo-Scientific, Waltham, Massachusetts, USA) were treated per wells and incubate for 10 min at 37 °C. Cells were then washed with PBS and observed under a fluorescence microscope.

To measure the levels of peroxisome-specific ROS, the pHyPer-PTS1 system was used [28]. Chang/HyPer-PTS1 cells were cultured and transferred to calcium-deficient medium for a predetermined time. Fluorescence in the cells was monitored under a fluorescence microscope.

### Calcium assay

Calcium levels were determined using the calcium assay kit (#ab102505 Calcium Colorimetric Assay Kit, Abcam, Cambridge, MA, USA), following the manufacturer’s protocol and normalized to protein content.

### Peroxisome isolation

Peroxisome were isolated using peroxisome isolation kit (#PEROX1-1KT Peroxisome Isolation Kit, Sigma-Aldrich, St. Louis, MO, USA), following the manufacturer’s protocol.

### Catalase activity assay

Catalase activity were determined using catalase activity kit (#83464 Catalase Activity Assay Kit Abcam, Cambridge, MA, USA), following the manufacturer’s protocol and normalized to protein content.

### siNBR1 transfection

Small interfering (si)RNA against mouse NBR1 was purchased from Bioneer (#17966-3, Bioneer, Daejeon, Republic of Korea. AML 12 cells were transfected with the siRNAs using Lipofectamine RNAiMAX Reagent (Invitrogen). After 24 h, the medium were replaced and harvested at 48 h after transfection.

### Statistical analysis

Data are presented as mean ± SD and originate from at least three different experiments. Differences between the groups were evaluated using either a two-tailed Student’s t-test or one-way analysis of variance, and *p* < 0.05 was considered statistically significant. Tukey HSD post-hoc test was conducted after the one-way ANOVA analysis.

## Results

### Calcium deficiency promotes peroxisomal protein degradation in a time-dependent manner

**Fig. 1:**
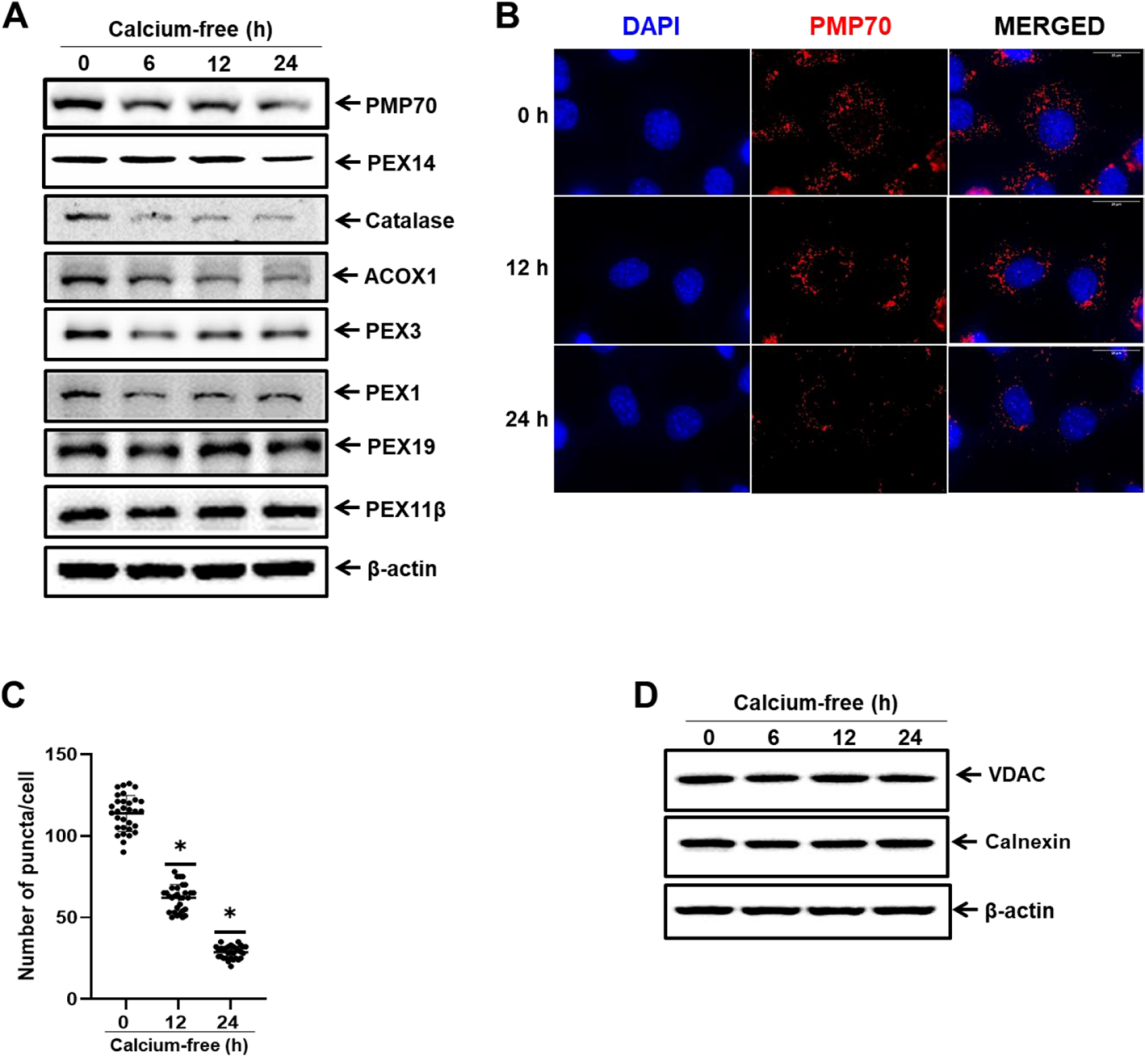
Calcium deficiency induces peroxisomal protein degradation in a time-dependent manner. **A** Immunoblot analysis of AML12 cells in calcium-deficient medium for indicated durations. Whole cell lysates were reacted with anti-catalase, anti-Pex14, anti-PMP70, anti-Acox1, anti-Pex3, anti-Pex1, anti-Pex19, anti-Pex11β, and anti-β-actin for protein expression analyses. **B** AML12 cells in calcium-deficient medium for indicated durations were fixed and immunostained with anti-PMP70 (red), shown as representative fluorescence image. Scale bar represents 25 µm. **C** Quantification of PMP70 puncta represents the number of peroxisomes per cell. Data are expressed as means ± S.D. (*n* = 3, independent experiments, 30 cells were analyzed in each experiment), * *p* < 0.05. **D** Immunoblot analysis of AML12 cells in calcium-deficient medium for indicated durations. Whole cell lysates were reacted with anti-VDAC, anti-Calnexin, and anti- β-actin

To investigate the significance of calcium at the subcellular organelle level, specifically in peroxisomes, we used an in-vitro model of calcium deficiency across several cell lines, including AML12 cells (Fig. 1). We assessed the protein expression of subcellular organelle markers, including those of peroxisomes, mitochondria, and ER at different time intervals after placing the cells in calcium-free media. The peroxisome membrane proteins PMP70 and Pex14, matrix proteins catalase and ACOX1, and biogenesis factors Pex1 and Pex3 demonstrated a marked, time-dependent decrease. However, there were no discernible changes in Pex19 and Pex11b proteins (Fig. 1A). Additionally, through immunofluorescence staining, we confirmed the reduction in peroxisomal protein puncta in calcium-deficient conditions in AML12 cells compared to those in the normal medium (Fig. 1B and C). However, protein expression levels of the mitochondria marker VDAC and the ER marker Calnexin remained unaltered (Fig. 1D).

To further validate the calcium deficit over various timeframes, we measured intracellular calcium levels at different time points using a calcium assay kit. The calcium content was significantly decreased in AML12 cells in a time-dependent manner (Additional file: Fig. S1A). The isolated peroxisomes also show significantly decreased calcium content (Additional file: Fig. S1C and D).We also confirmed that intracellular calcium level affects peroxisomal protein degradation by rescuing the degradation of peroxisomal proteins (Additional file: Fig. S1B). These results indicate that calcium-deficit condition encourages the breakdown of peroxisomal proteins.

### Calcium deficiency abates catalase activity and increases ROS accumulation

**Fig. 2:**
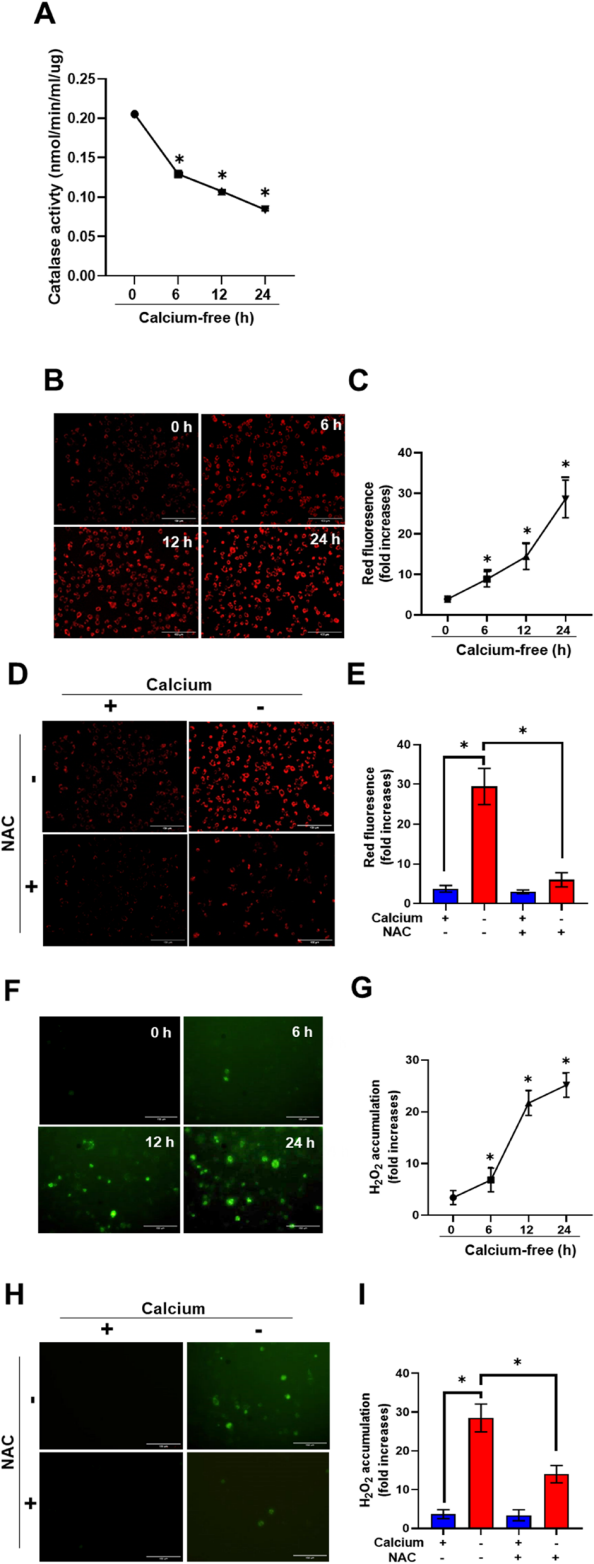
Calcium deficiency abates catalase activity and increases ROS accumulation. **A** Catalase activity assay of AML 12 cells in calcium deficient medium for indicated durations. **B** AML12 cells in calcium-deficient medium for indicated durations were stained with Cell ROX. Red fluorescence represents the ROS. Scale bar represents 150 µm. **C** Quantification of relative red fluorescence intensity indicates ROS content. Data are expressed as means ± S.D. (*n* = 3, independent experiments, 30 cells were analyzed in each experiment), * *p* < 0.05. **D** AML12 cells in calcium-deficient medium for indicated durations treated with antioxidant NAC were stained with Cell ROX. Scale bar represents 150 µm. **E** Quantification of relative red fluorescence intensity indicates ROS content. Data are expressed as means ± S.D. (*n* = 3, independent experiments, 30 cells were analyzed in each experiment), * *p* < 0.05. **F** Chang Hyper-SKL cells in calcium deficient medium for indicated durations. Green fluorescence represents ROS content from peroxisomes. **G** Quantification of green fluorescence intensity indicates peroxisomal ROS content. Data are expressed as means ± S.D. (*n* = 3, independent experiments, 30 cells were analyzed in each experiment), * *p* < 0.05. **H** Chang Hyper-SKL cells in calcium deficient medium treated with antioxidant NAC for indicated durations. Green fluorescence represents the ROS content in peroxisomes. Scale bar represents 150 µm. **I** Quantification of green fluorescence intensity indicates peroxisomal ROS content. Data are expressed as means ± S.D. (*n* = 3, independent experiments, 30 cells were analyzed in each experiment), * *p* < 0.05

We speculated that the degradation of peroxisomal proteins with dropping calcium levels could be attributed to ROS accumulation. We analyzed catalase activity in calcium-deficient conditions (Fig. 2A). Intracellular ROS production was measured using Cell ROX and quantified. ROS production increased as a result of calcium deficiency (Fig. 2B and C). ROS accumulation was demonstrated by treating the cells with antioxidant NAC (5 mM). NAC treatment suppressed the relative fluorescent intensity (Fig. 2D and E).

To verify that ROS accumulated in peroxisomes, we used Chang Hyper-SKL cells (a fluorescent sensor for detection of cellular H_2_O_2_ in peroxisome) and observed fluorescence during calcium-depleted conditions. ROS was indeed accumulated in peroxisomes as calcium levels dropped (Fig. 2F and G). We further validated ROS accumulation in peroxisome by using Chang Hyper-SKL cells with NAC (5 mM). NAC treatment suppressed the relative fluorescent intensity (Fig. 2H and I).

These findings imply that calcium deficit reduces catalase activity and causes ROS buildup in peroxisomes.

### Calcium deficiency decreases mTORC1 activity, activating TFEB

**Fig. 3:**
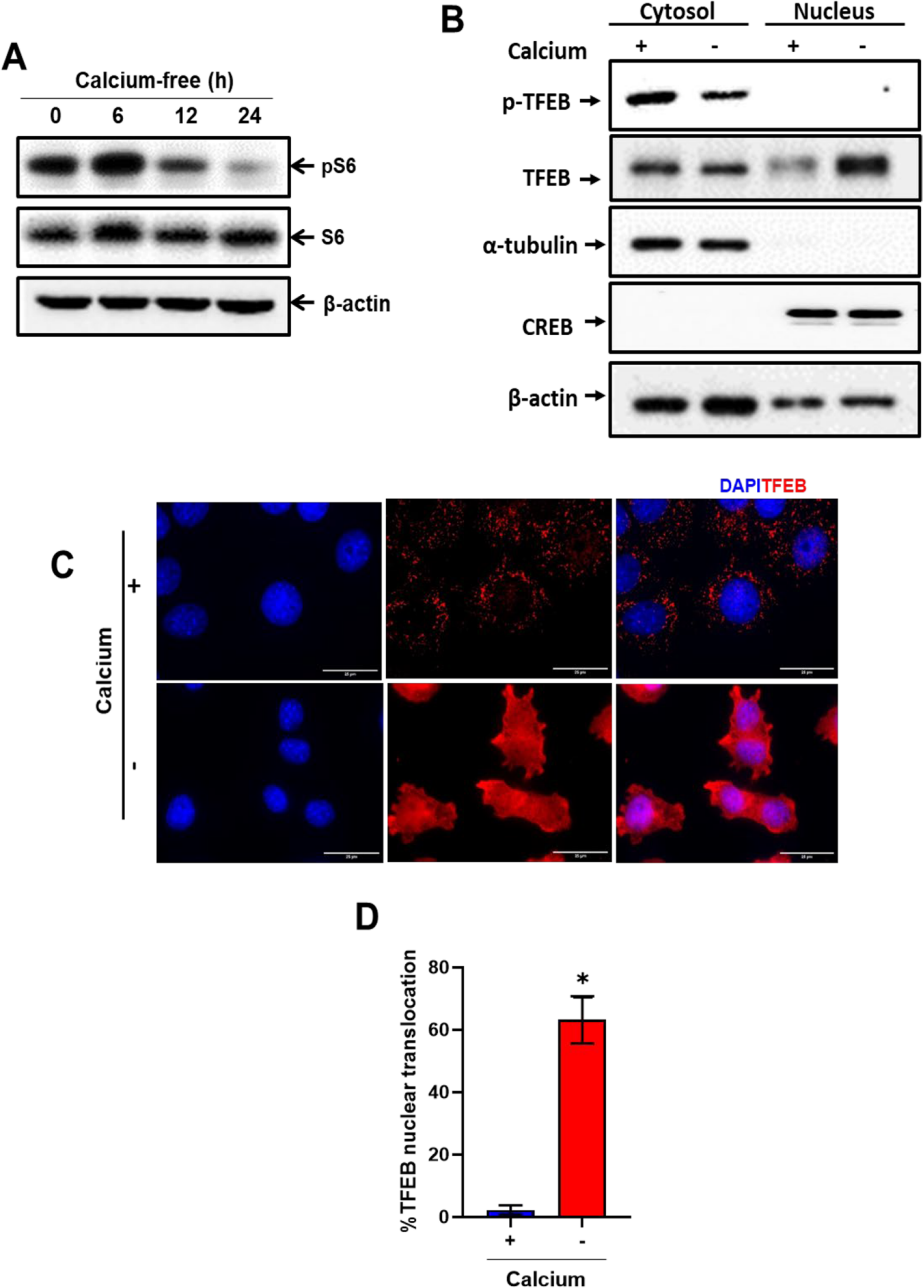
Calcium deficiency suppresses mTORC1 activity, activating TFEB. **A** Immunoblot analysis of AML12 cells in calcium-deficient medium for indicated durations. Whole cell lysates were reacted with anti-phosphoS6, anti-S6, and anti-β-actin. **B** Cytosolic and nuclear fractions from AML12 cells with or without calcium for 24 h. Immunoblot fractions with anti-TFEB and p-TFEB (Ser211). For the cytosolic and nuclear fractions, alpha-tubulin and CREB antibodies were utilized as the controls for cytosolic and nuclear fractionations, respectively. **C** Immunofluorescence of AML12 cells with or without calcium with anti-TFEB (red). Scale bar represents 25 µm. **D** Quantification of red fluorescence in the nucleus indicates activation of TFEB. Data are expressed as means ± S.D. (*n* = 3, independent experiments, 30 cells were analyzed in each experiment), * *p* < 0.05

To evaluate mTORC1 activity in conditions of calcium deficiency, we examined the phosphorylation of S6. As depicted in Fig. 3A, the phosphorylation of S6 exhibited a marked decrease in a time-dependent manner.

We subsequently examined the translocation of TFEB from cytosol to nucleus. Employing cellular fractionation, we isolated pure nuclear and cytosolic fractions. In calcium-deficient conditions, TFEB demonstrated a significant translocate in the nucleus compared to that in the normal medium and p-TFEB (Ser211) expression was decreased (Fig. 3B). Additionally, we confirmed the translocation of TFEB in the nucleus through anti-TFEB immunofluorescence during calcium-deficient conditions compared to control (Fig. 3C and D). We further validated the reduction of S6 phosphorylation in RPE-1 cells under calcium-deficient conditions (Additional file: Fig. S3A). These data suggest that mTORC1 inactivation, coupled with ROS accumulation, activates TFEB in calcium-deficient conditions.

### Calcium deficiency induces selective autophagy in peroxisomes

**Fig. 4:**
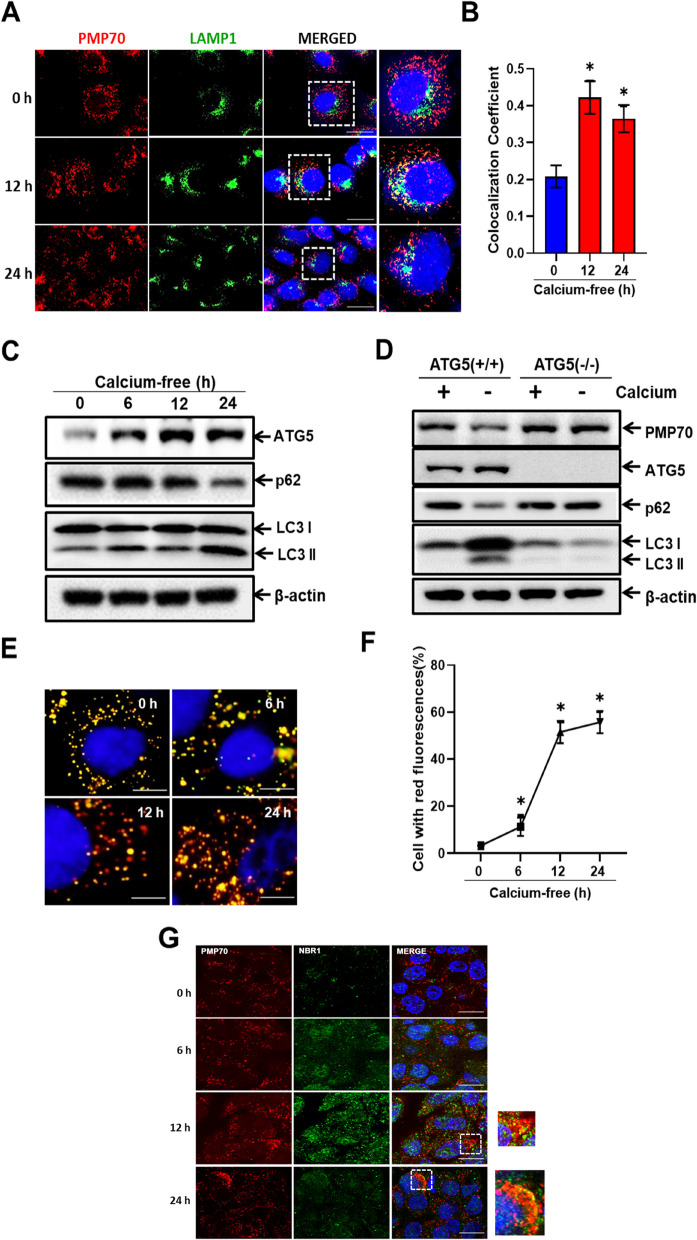
Calcium deficiency induces selective autophagy in peroxisomes. **A** AML12 cells in calcium-deficient medium for indicated durations, immunostained with anti-PMP70 (red) and anti-LAMP1 (green), shown as representative fluorescence image. Scale bar represents 25 µm. **B** Quantification of PMP70 and LAMP1 co-localization. Data are expressed as means ± S.D. (*n* = 3, independent experiments, 30 cells were analyzed in each experiment), * *p* < 0.05. **C** Immunoblot analysis of AML12 cells in calcium-deficient medium for indicated durations. Whole cell lysates were reacted with anti-ATG5, anti-p62, anti-LC3II, and anti-β-actin. **D** Immunoblot analysis of ATG5 (+ / +) and ATG5 (-/-) MEF cells in calcium-deficient medium for 24 h. Whole cell lysates were reacted with anti-PMP70, anti-ATG5, anti-p62, anti-LC3II, and anti-β-actin. **E** Immunofluorescence of mRFP-GFP-SKL cells in calcium-deficient medium for indicated durations. Scale bar represents 25 µm. **F** Quantification of red fluorescence represents pexophagy. Data are expressed as means ± S.D. (*n* = 3, independent experiments, 30 cells were analyzed in each experiment), * *p* < 0.05. **G** RPE-1 cells in calcium deficient medium for indicated durations were fixed and immunostained with anti-PMP70 (red) and anti-NBR1 (green) shown as representative fluorescence image. Scale bar represents 25 µm

To investigate the effect of calcium deficiency in peroxisomal degradation through lysosomal co-localization, we performed anti-PMP70 (red) and anti-LAMP1 (green) immunofluorescence staining. The intensity of peroxisome- and lysosome co-localization increased at 12 h and 24 h after replacing the media with calcium-free media (Fig. 4A and B) whereas chloroquine treatment inhibited co-localization (Additional file: Fig. S5). Next, to evaluate the autophagic flux in calcium-deficient conditions, we examined the protein expression levels of autophagy related proteins such as ATG5, p62, and LC3II using western blot analysis. As shown Fig. 4C, ATG5 and LC3II expression increased in a time-dependent manner in calcium-deficient conditions. However, p62 expression decreased (Additional file: Fig. S7A).

To further confirm the increase in autophagic flux as calcium levels dropped, we used autophagy related gene 5, ATG5 (+ / +), and ATG5 (-/-) MEF cells in calcium-deficient conditions. In ATG5 (+ / +) MEFs, autophagic flux was induced, resulting in decreased p62 and increased LC3II expression. However, ATG5 (-/-) cells did not exhibit detectable autophagic flux in calcium-deficient conditions. Furthermore, the expression of peroxisomal marker protein PMP70 was significantly decreased in ATG5 (+ / +) but remained unchanged in ATG5 (-/-) MEF cells (Fig. 4D and Additional file: Fig. S7B).

We then evaluated the increase in autophagic flux leading to selective autophagy in peroxisomes using RPE-1 cells expressing the mRFP-GFP-SKL plasmid. When peroxisomes and lysosomes fuse, acid-sensitive GFP fluorescence vanishes, while the acid-insensitive mRFP signal remains [29]. We observed a significant increase in red puncta as the calcium levels dropped, indicating pexophagy in a time-dependent manner (Fig. 4E and F). Furthermore, the increase of the autophagic flux at RPE-1 and HepG2 cells in calcium-deficient conditions was confirmed (Additional file: Fig. S4A and B). Additionally, we speculated the degradation of peroxisomes through pexophagy involving NBR1. To investigate this, we performed immunofluorescence staining in RPE-1 cells using anti-PMP70 (red) and anti-NBR1 (green) under calcium-deficient conditions for a predetermined time. We observed an increased co-localization between PMP70 and NBR1 as calcium levels dropped (Fig. 4G). Taken together, these findings indicate that calcium deficiency increased autophagic flux for peroxisome-specific degradation.

### Calcium deficiency-induced pexophagy is regulated by ROS-mediated TFEB activation

**Fig. 5:**
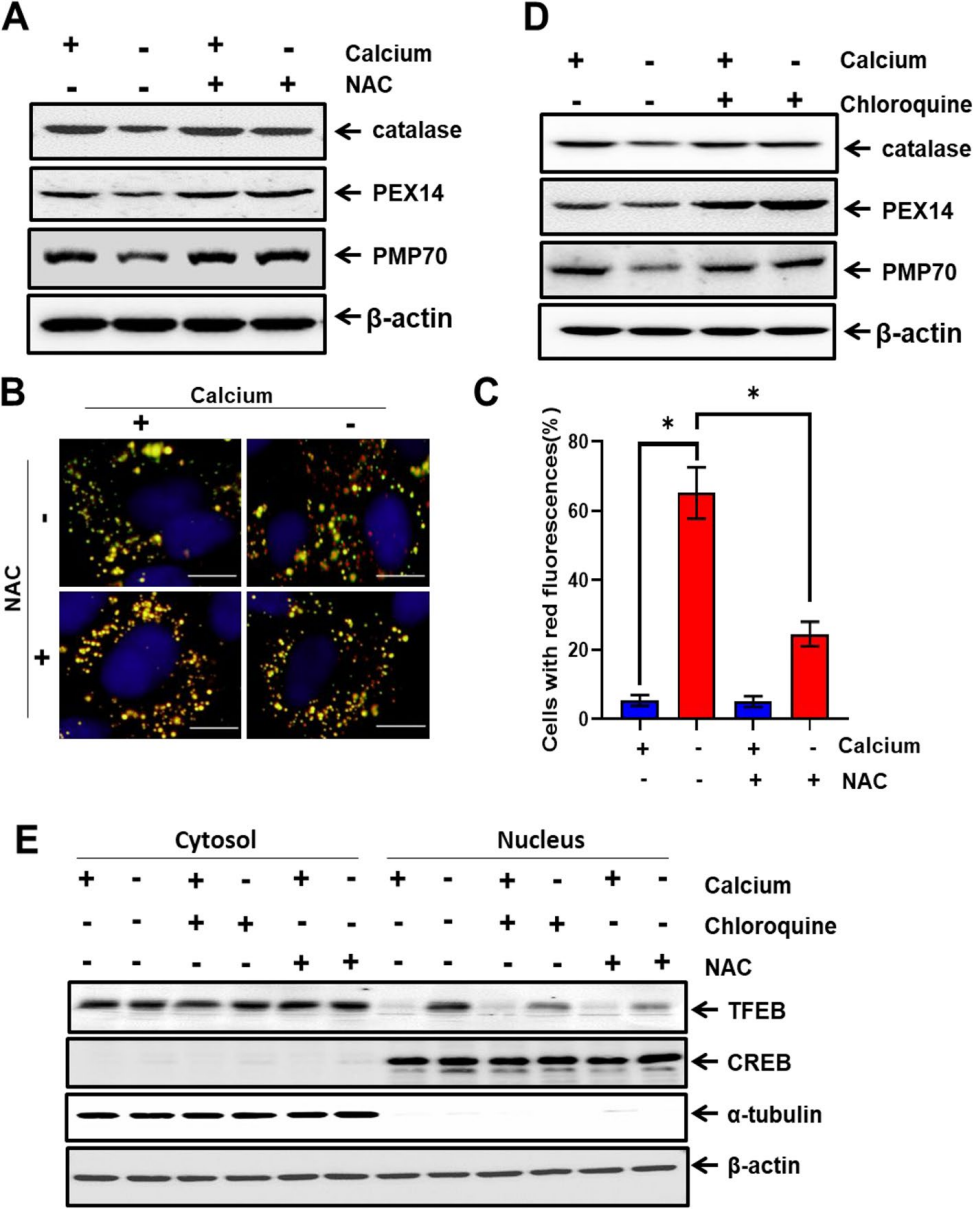
Calcium deficiency-induced pexophagy is regulated by ROS-mediated TFEB activation. **A** Immunoblot analysis results of AML12 cells in calcium-deficient medium with or without NAC treatment. Whole cell lysates were reacted with anti-catalase, anti-Pex14, anti-PMP70, and anti-β-actin. **B** Immunofluorescence of mRFP-GFP-SKL cells in calcium-deficient medium for indicated durations with or without NAC treatment. Scale bar represents 25 µm. **C** Quantification of red fluorescence represent pexophagy. Data are expressed as means ± S.D. (*n* = 3, independent experiments, 30 cells were analyzed in each experiment), * *p* < 0.05. **D** AML12 cells in calcium-deficient medium with or without chloroquine treatment are immunoblotted. Whole cell lysates were reacted with anti-catalase, anti-Pex14, anti-PMP70, and anti-β-actin. **E** Cytosolic and nuclear fractions from AML12 cells with or without calcium for 24 h. Also includes treatment of NAC and chloroquine. Immunoblot the fractions with anti-TFEB. For the cytosolic and nuclear fractions, alpha-tubulin and CREB antibodies, respectively, were utilized as the controls

To confirm whether ROS accumulation, induced by calcium deficiency, regulates pexophagy, we treated AML12 cells in calcium-deficient conditions with the antioxidant NAC (5 mM) and the autophagy inhibitor chloroquine (5 µM). We then assessed the protein expression of peroxisomal markers using western blot analysis. Calcium deficiency decreased the protein expression of catalase, Pex14, and PMP70, and was mitigated by NAC treatment (Fig. 5A). Additionally, red puncta of RPE-1 cells expressing the mRFP-GFP-SKL plasmid under calcium deficiency reduced with NAC treatment compared to that without NAC (Fig. 5B and C).

To further corroborate the autophagic destruction of the peroxisome, we examined the expression of peroxisomal proteins after treatment with the autophagy inhibitor chloroquine. Calcium deficiency decreased the protein expression of catalase, Pex14, and PMP70, and this effect was rescued by treatment with chloroquine (Fig. 5D).

Based on these results, we speculated that pexophagy occurs because of TFEB translocation. Treatment with NAC (5 mM) or chloroquine (5 µM) may inhibit translocation of TFEB in calcium deficient condition. To verify this, the nuclear and cytosolic fractions were purely isolated and the translocation of TFEB was evaluated. As shown in Fig. 5E, TFEB was translocated to the nucleus in response to calcium deficiency; however, treatment with NAC and chloroquine markedly protected TFEB translocation in calcium-deficient conditions (Fig. 5E and Additional file: Fig. S8C).

## Discussion

The results from our study imply a connection between calcium and autophagy of peroxisome that is mediated by the regulation of TFEB and ROS signaling. Calcium, known for its role in maintaining tissue rigidity, strength, and flexibility, aid in the formation of bones and teeth, facilitating physiological movement. The ER has been recognized as the storage site and channel for calcium oscillations within individual cells. Other cellular organelles such as lysosome and mitochondria can receive calcium through ER calcium channels, but there is no conclusive evidence that ER-peroxisome channels exist [30].

The calcium content in the peroxisome matrix of resting cells is 20 times higher than that in the cytoplasm, indicating calcium's role in controlling peroxisome metabolism [4]. In addition, once intracellular calcium stores are depleted and calcium influx occurs across the plasma membrane, peroxisomes were found to absorb calcium in Hela cells [31]. In our study, we demonstrated that calcium deficiency induced the degradation of peroxisomal proteins (Fig. 1) which open the door to further study calcium in particular subcellular organelles. We observed no decrease in protein expression of Pex19 and Pex11b in calcium deficiency condition which might be feedback loop of counterbalance against the pexophagy flux [32].

Previously, we reported the involvement of ROS in hepatic injury during prolonged fasting and pexophagy under catalase-deficient conditions [26, 33]. Peroxisomes are highly sensitive organelles involved in lipid and redox metabolism. In addition, they exchange lipid metabolites and ROS to communicate [6]. In this study, we showed that depletion of calcium diminishes catalase activity, leading to ROS buildup in the peroxisomes (Fig. 2).

By integrating anabolic and catabolic activities with food, energy, and oxygen availability, as well as growth-factor signaling, mTOR controls cellular and organismal homeostasis [13]. When under oxidative stress, mTORC1 is inhibited and autophagy is triggered in response to nutrient deprivation and reduced growth-hormone levels [14]. The level of phosphorylation of S6 controlled autophagic and protein production fluxes in isolated rat hepatocytes. Autophagy is inhibited by the phosphorylation of S6, suggesting that autophagy occurs when S6 phosphorylation levels are reduced [34]. We also observed decreased mTORC1 activity as the S6 phosphorylation decreased with calcium depletion (Fig. 3A), indicating that calcium levels can regulate the mTORC1 signaling pathway.

The mTORC1 and TFEB co-localize on the lysosome membrane. TFEB is inhibited by mTORC1 in the presence of nutrients. In contrast, pharmacological suppression of mTORC1, malnutrition, and lysosomal disruption activate TFEB by encouraging its nuclear translocation [16]. When there is mitochondrial oxidative stress, TFEB is translocated to the nucleus, leading to selective autophagy, such as mitophagy, for the functional adaptation of pancreatic beta-cells to metabolic stress [3]. Here, we observed the peroxisome’s selective destruction and ROS build-up together with the translocation of TFEB to the nucleus during calcium deprivation. These results suggest that pexophagy occurs through ROS-mTORC1-TFEB pathway to eliminate peroxisomal oxidative stress. Additionally, extreme calcium deprivation may cause apoptosis. Furthermore, validating the transcriptional activity of TFEB, we detected an increase in protein expression of target genes such as LAMP1 and Cathepsin C (Additional file: Fig. S3B).

The well-known autophagy inhibitor chloroquine prevents the fusion of lysosomes and autophagosomes, inhibiting the breakdown of proteins in lysosomes [35]. In addition, chloroquine is known to boost the quantity, import, and biochemical activity of peroxisomes, which improves cell survival by inhibiting autophagic pathways in the growth medium [36]. As a result, chloroquine may prevent TFEB from translocating to the nucleus, preventing the peroxisome degradation necessary for maintaining redox equilibrium. Antioxidants like NAC can also prevent TFEB translocation to the nucleus, particularly when there is no oxidative stress. In our study, we found that both chloroquine and NAC attenuated calcium deficiency-induced pexophagy (Fig. 5), thus corroborating our findings.

Despite mitochondria producing excessive ROS, potentially owing to fatty-acid oxidation, and despite the necessity of calcium for mitochondrial function, we found no signs of the induction of mitophagy. In calcium deficiency conditions, the expression of oxidative phosphorylation-related mitochondrial proteins tended to increase, indicating that the antioxidant capabilities of mitochondria may adequately guard against oxidative damage resulting from decreased catalase activity (Additional file: Fig. S9A).

Taken together, our results demonstrate that calcium deficiency leads to the degradation of peroxisomal proteins. We demonstrated the pivotal role of calcium in physiological or pathological conditions and its impact on peroxisomes in maintaining cell survival. Calcium directly affect catalase activity, a crucial function of peroxisomes. Our findings are consistent with those of previous studies on the effects of calcium on catalase and superoxide dismutase (SOD). As calcium concentration increases, catalase activity increases while SOD activity decreases [37].

Furthermore, mTORC1 and TFEB involvement indicated lysosomal interaction during calcium-deficient conditions. Thus, we hypothesize that peroxisomes and lysosomes play crucial roles in calcium homeostasis, among other functions. Further studies are needed to gain a detailed understanding of the importance of calcium in peroxisome functions, as well as in different disease conditions.

Our data also suggest the presence of calcium channels in peroxisomes, similar to those in other cellular organelles. In addition, we can hypothesize peroxisomes may store calcium and release it when the calcium levels drop or in response to stress, as in to how lysosomes release in response to oxidative stress [3].

## Conclusion

**Fig. 6:**
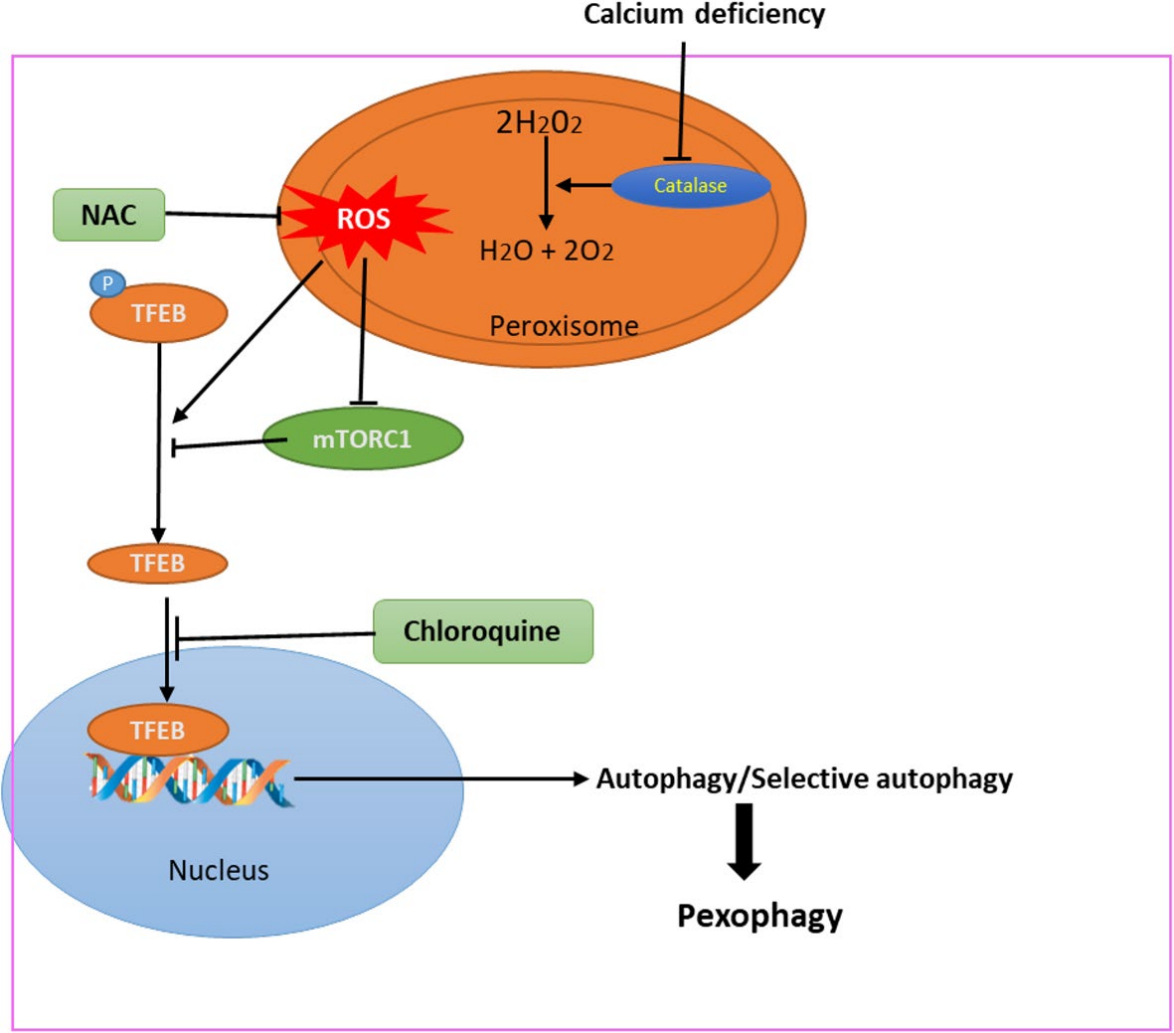
Schematic representation of role of calcium in peroxisome. Calcium deficiency increases ROS accumulation by inhibiting catalase activity in peroxisomes and reduces phosphorylation of TFEB by inhibiting mTORC1 activity. Combining ROS accumulation, TFEB translocates to nucleus for autophagic degradation of peroxisome. Hence, pexophagy is induced by a calcium deficit via the ROS-mTORC1-TFEB pathway

Thus, the effect of calcium on catalase activity resulted in ROS accumulation. This ROS accumulation triggers pexophagy through the ROS-mTORC1-TFEB pathway. This activation of TFEB induces selective autophagic degradation of peroxisomes, facilitating clearance of damaged peroxisomes and removal of excessive ROS (Fig. 6). Thus, the study clearly revealed the importance of calcium in peroxisomal function and eradication of oxidative stress. We also conclude that peroxisomes are substantially more susceptible to calcium homeostasis.

### Supplementary Information

**Supplementary Material 1.**

**Supplementary Material 2.**

## Data Availability

Data sharing is not applicable to this article as no datasets were generated or analyzed during the current study.

## Abbreviations

AML12: Alpha mouse liver 12
ATG: Autophagy related gene
ATM: Ataxia-telangiectasia mutated
Ca^2+^: Calcium ions
CREB: CAMP response element-binding protein
DMEM: Dulbecco's Modified Eagle Medium
ER: Endoplasmic reticulum
FBS: Fetal bovine serum
GFP: Green fluorescent protein
HepG2: Hepatoma G2
INS: Insulin
LAMP1: Lysosomal associated membrane protein 1
LC3II: Light chain 3B
MEF: Mouse embryonic fibroblast
mTOR: Mammalian target of rapamycin
mTORC1: Mammalian target of rapamycin complex 1
NAC: N-acetylcysteine
NBR1: Neighbor of BRCA1 gene 1 protein
Pex1: Peroxisomal biogenesis factor 1
Pex2: Peroxisomal biogenesis factor 2
Pex5: Peroxisomal biogenesis factor 5
Pex10: Peroxisomal biogenesis factor 10
PEX14: Peroxisomal biogenesis factor 14
PMP70: 70-kDa Peroxisomal membrane protein
p62: Ubiquitin binding protein p62
PBS: Phosphate-buffered saline
PBD: Peroxisome biogenesis disease
PTS1: Peroxisome targeting signal 1
pS6: Phosphorylation of ribosomal protein S6
S6: Ribosomal protein S6
RFP: Red fluorescent protein
RIPA: Radio-immunoprecipitation assay buffer
ROS: Reactive oxygen species
RPE-1: Human retinal pigment epithelial-1
SOD2: Superoxide dismutase
TFEB: Transcription factor EB
UBXD8: Ubiquitin-like (UBX)-domain-containing protein
VDAC: Voltage dependent anion channel protein 1
