# Supplementary material for: TFEB activation triggers pexophagy for functional adaptation during oxidative stress under calcium deficient-conditions
Source: Cell Commun Signal. 2024 Feb 21;22:142. doi: 10.1186/s12964-024-01524-x (PMC10880274; doi:10.1186/s12964-024-01524-x)
Supplement: Supplementary file 1 — Supplementary Material 1. [file 12964_2024_1524_MOESM1_ESM.docx]

**Additional file 1**

# **TFEB activation triggers pexophagy for functional adaptation during oxidative stress under calcium deficient-conditions**

# Laxman Manandhar^1¶^, Raghbendra Kumar Dutta^1#¶^, Pradeep Devkota^1^, Arun Chhetri^1^, Xiaofan Wei^1^, Channy Park^1^, Hyug Moo Kwon^2^, and Raekil Park^1^*

^1^ Department of Biomedical Science and Engineering, Gwangju Institute of Science and Technology, Gwangju 61005, Republic of Korea

^2^ School of Life Sciences, Ulsan National Institute of Science and Technology, Ulsan, Republic of Korea

* Correspondence:

# Raekil Park, M.D., Ph.D.

# Department of Biomedical Science & Engineering,

# Gwangju Institute of Science and Technology, Gwangju 61005, Republic of Korea

# Tel.: +82-62-715-5361; Fax: +82-62-715-5309; E-mail: rkpark@gist.ac.kr

# ^¶^These authors contributed equally.

# # Current address: Department of Chemistry (Biochemistry Division) Crosley Tower, University of Cincinnati, Ohio, 45221 USA

# **
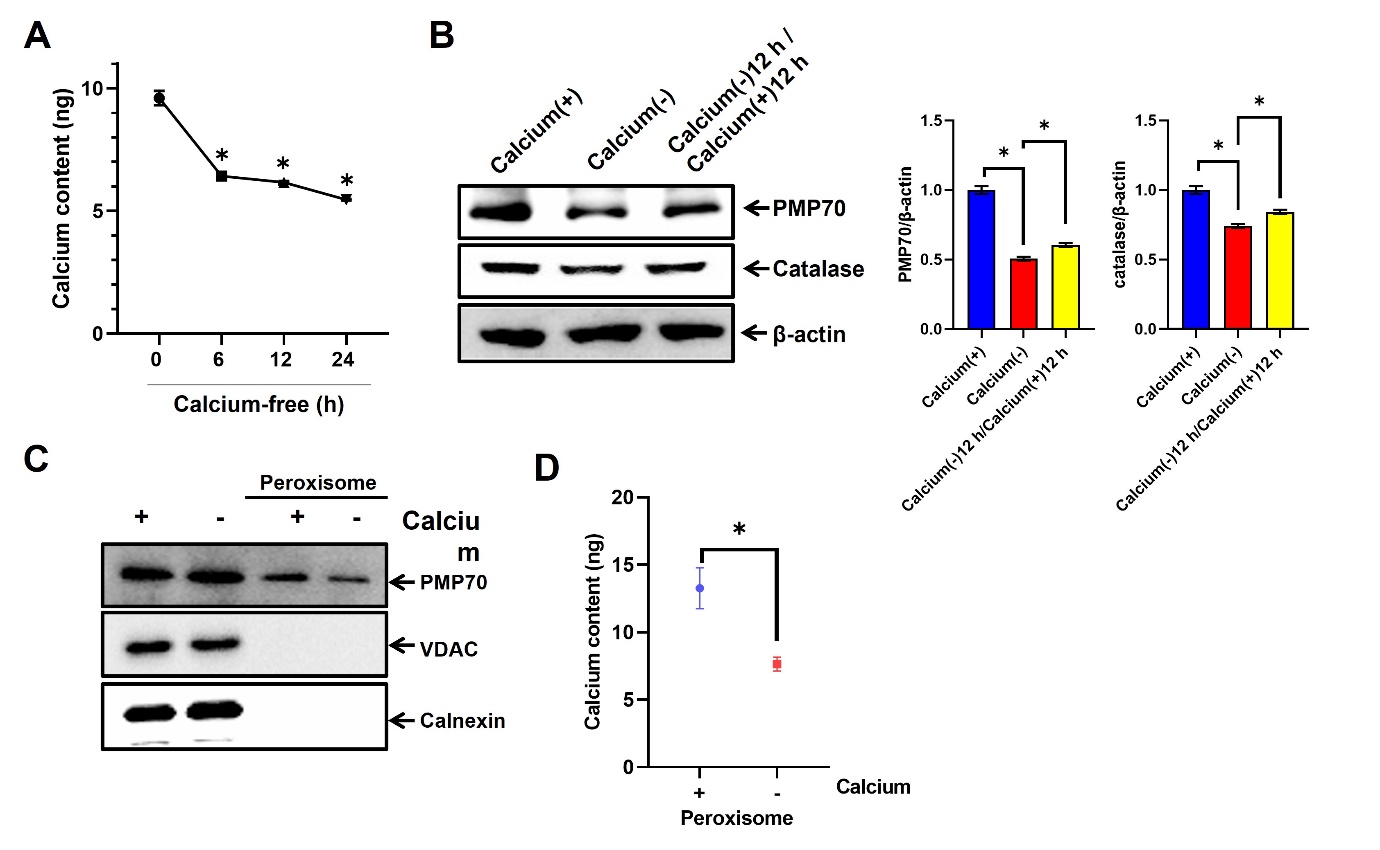
**

**Fig. S1 Calcium level affects peroxisomal protein degradation.**

(A) Calcium assay of AML12 cells in calcium deficient medium for indicated durations. Data are expressed as means ± S.D. (n=3, independent experiments), * *p* < 0.05. (B) Immunoblot analysis of AML 12 cell where calcium (+) indicate the use of normal medium, calcium (-) indicate calcium deficient medium for 24 h, and calcium(-)12 h/calcium(+)12 h indicate calcium deficient medium for 12 h replaced later with calcium medium for 12 h and their respective quantification to the right against β-actin. Data are expressed as means ± S.D. (n=3, independent experiments), * *p* < 0.05. (C) Peroxisomal fractionation and Immunoblot analysis of AML 12 cells with or without calcium in media. (D) Calcium contents in peroxisomal fraction of AML 12 cells with or without calcium in media normalized to protein content. Data are expressed as means ± S.D. (n=3, independent experiments), * *p* < 0.05.

# **
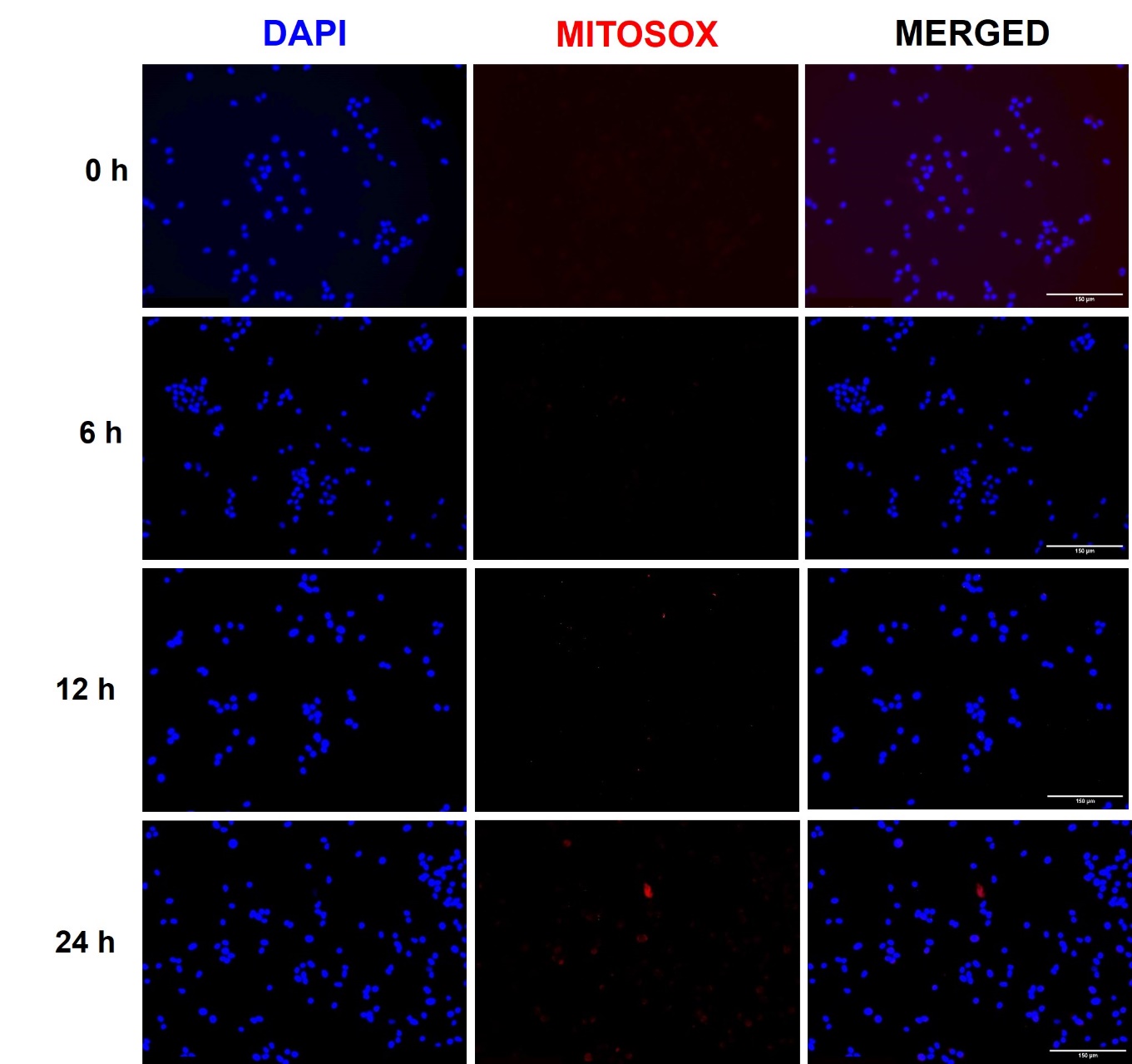
**

**Fig. S2. Calcium deficiency does not affect generation of mitochondrial ROS in AML12 cells.**

AML12 cells in calcium-deficient medium for indicated durations were stained with Mitosox. Red fluorescence represents the ROS in mitochondria. Scale bar represents 25 µm.

# **
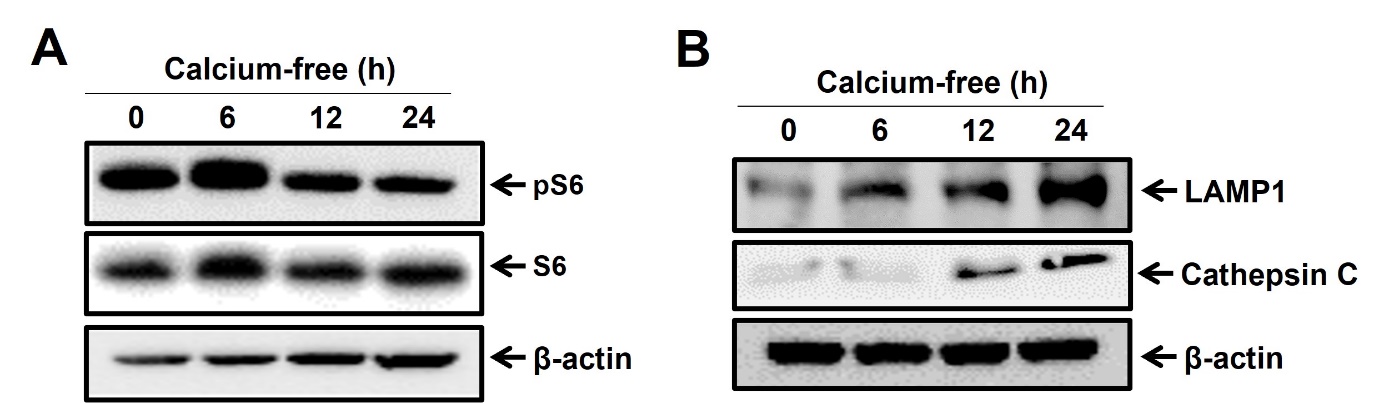
**

**Fig. S3. Calcium deficiency regulates activity of mTORC1 and expression of target proteins of TFEB in RPE-1 cells.**

(A) Immunoblot analysis of RPE-1 cells in calcium deficient medium for indicated durations. Whole cell lysates were reacted with anti-phosphoS6, anti-S6 and anti-β-actin. (B) Immunoblot analysis of AML 12 cells in calcium deficient medium for indicated period. Whole cell lysates were reacted with anti-LAMP1, anti-Cathepsin C, and anti-β-actin**.**

# **
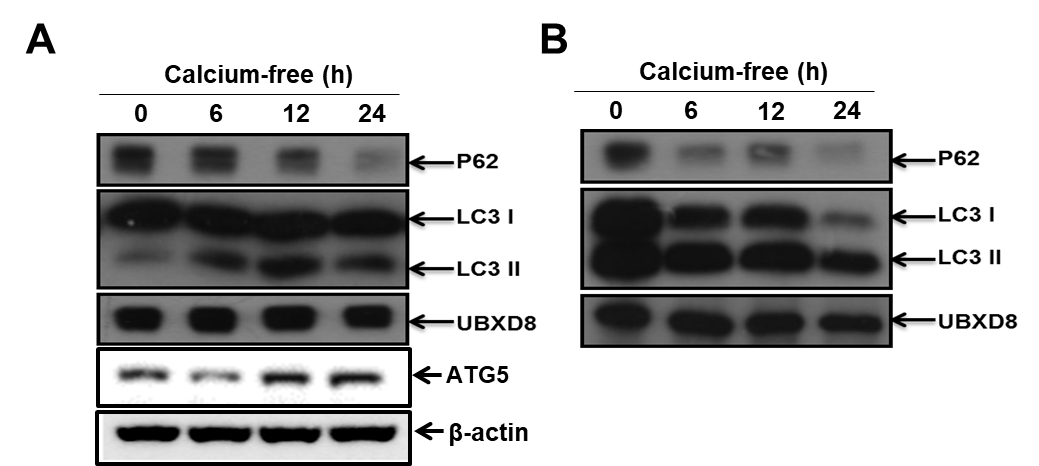
**

**Fig. S4. Calcium deficiency induces selective autophagy in RPE1 and HepG2 cells.**

(A) Immunoblot analysis of RPE-1 cells in calcium deficient medium for indicated durations. Whole cell lysates were reacted with anti-p62, anti-LC3II, anti-UBXD8, anti-ATG5, and anti-β-actin. (B) Immunoblot analysis of HepG2 cells in calcium deficient medium for indicated durations. Whole cell lysates were reacted with anti-p62, anti-LC3II, and anti-UBXD8.

**
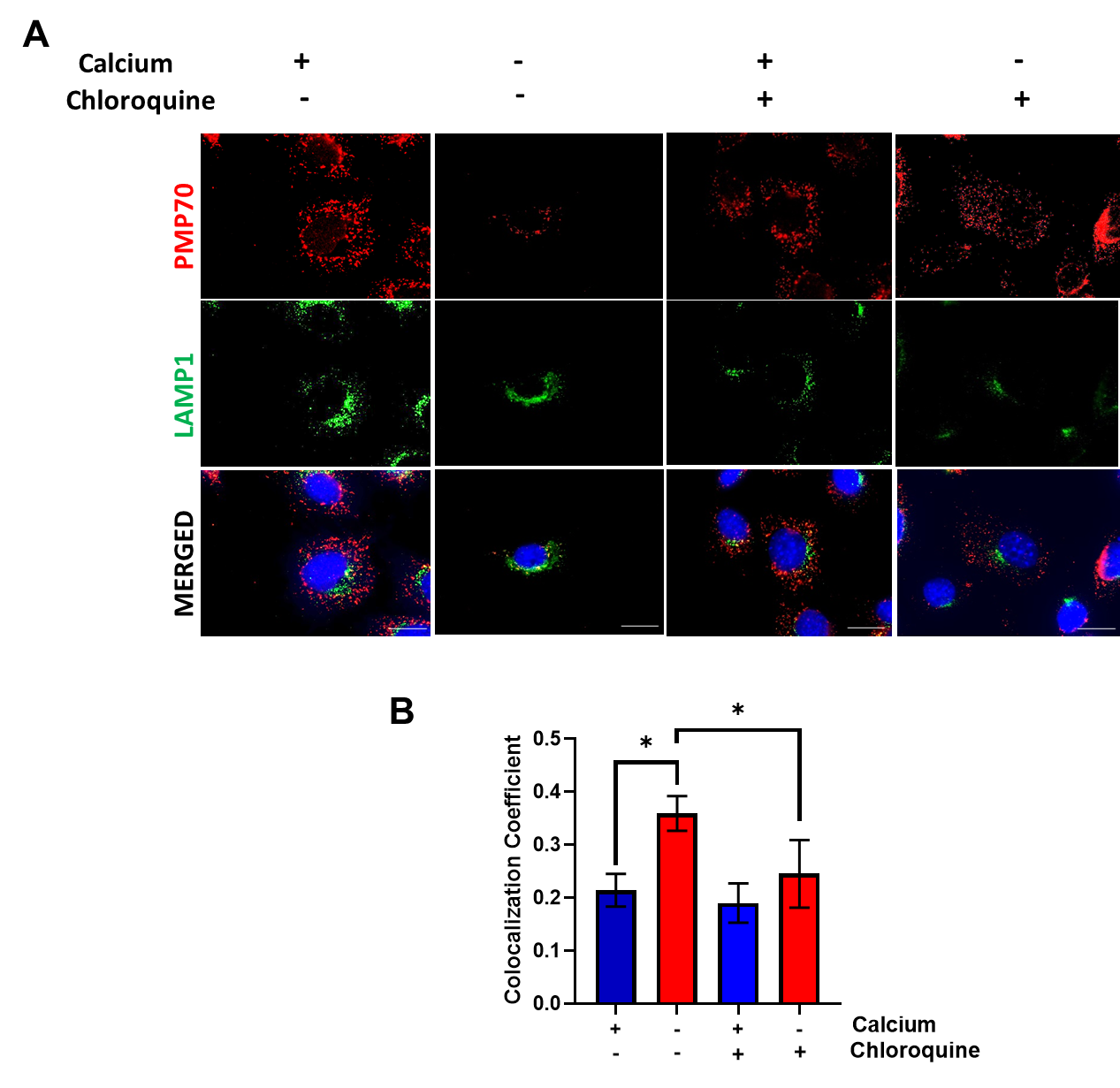
**

**Fig. S5. Chloroquine decreases PMP70 and LAMP1 co-localization in calcium deficiency.**

(A) AML12 cells in calcium-deficient medium, with or without chloroquine immunostained for PMP70 (red) and LAMP1 (green), shown as representative fluorescence images. Scale bar represents 25 µm. (B) Quantification of PMP70 and LAMP1 co-localization. Data are expressed as means ± S.D. (n=3, independent experiments, 30 cells were analyzed in each experiment), * *p* < 0.05.

**
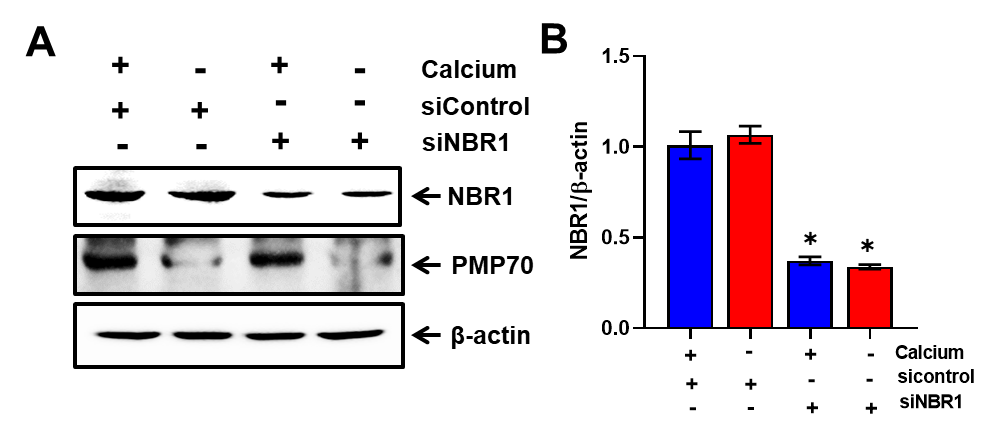
**

**Fig. S6. Silencing NBR1 did not rescue pexophagy in calcium deficiency.**

(A) Immunoblot analysis of AML 12 cells in calcium deficient medium with or without silencing NBR1. Whole cell lysates were reacted with anti-NBR1, anti-PMP70 and anti-β-actin. (B) Graphical representation knockdown efficiency of siNBR1. Protein expression was analysed against β-actin.

**
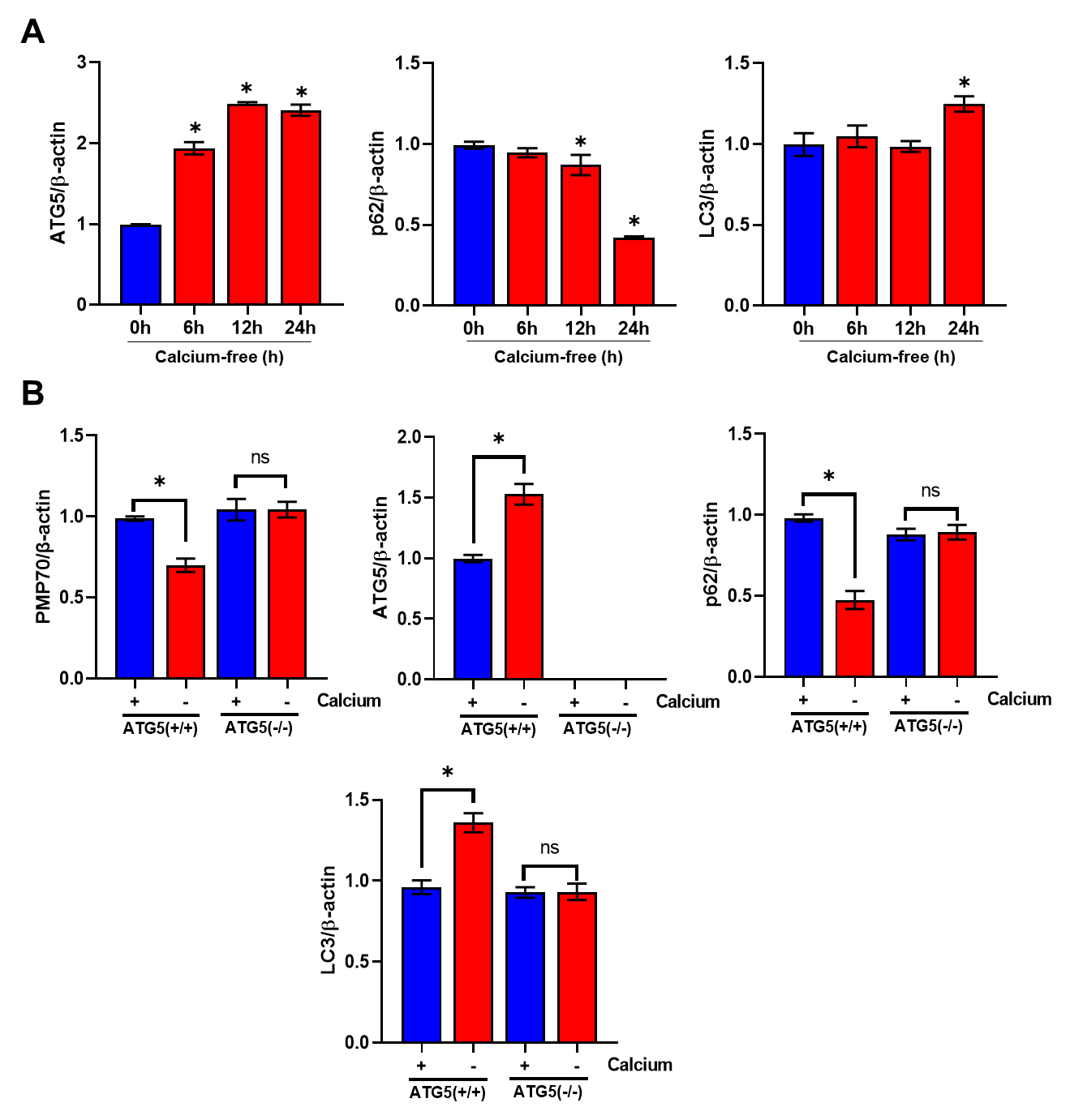
**

**Fig. S7. Protein Quantification.**

1. Quantification of protein expression of ATG5, p62 and LC3 against β-actin of AML 12 cells in calcium deficiency condition.
2. Quantification of protein expression of PMP70, ATG5, p62 and LC3 against β-actin in ATG5 wild type and ATG5 knockout MEF cells against β-actin with or without calcium.

**
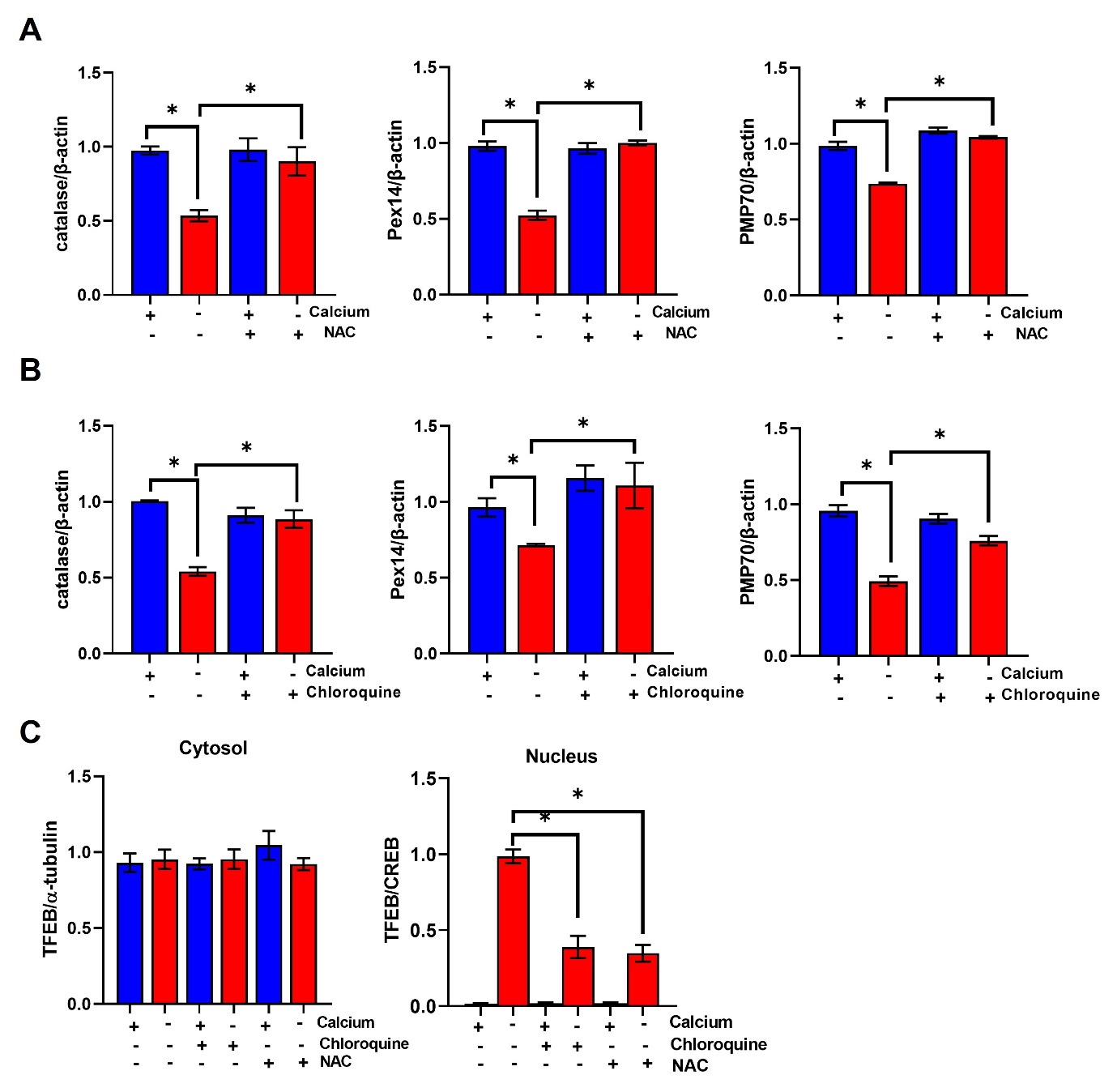
**

**Fig. S8. Protein Quantification**

1. Quantification of protein expression of catalase, Pex14 and PMP70 against β-actin of AML 12 cells in calcium deficient medium with or without NAC treatment.
2. Quantification of protein expression of catalase, Pex14 and PMP70 against β-actin of AML 12 cells in calcium deficient medium with or without chloroquine treatment.
3. Quantification of protein expression of TFEB against α-tubulin in cytosolic fraction and against CREB in nuclear extract in calcium deficiency with treatment of NAC and chloroquine.

**
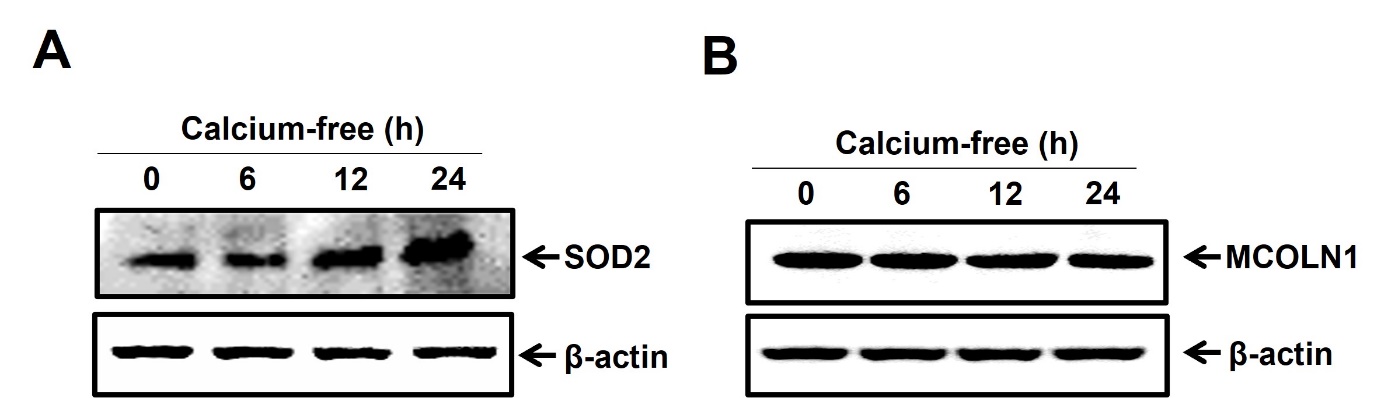
**

**Fig. S9. Calcium deficiency increases expression of SOD2 protein in AML12 cells and no change in MCOLN1 protein.**

Immunoblot analysis of AML12 cells in calcium deficient medium for indicated durations. Whole cell lysates were reacted with anti-SOD2 (A), anti-MCOLN1(B) and anti-β-actin.
