# Supplementary material for: TFEB activation triggers pexophagy for functional adaptation during oxidative stress under calcium deficient-conditions
Source: Cell Commun Signal. 2024 Feb 21;22:142. doi: 10.1186/s12964-024-01524-x (PMC10880274; doi:10.1186/s12964-024-01524-x)
Supplement: Supplementary file 2 — Supplementary Material 2. [file 12964_2024_1524_MOESM2_ESM.docx]

**Supplementary files**

# **TFEB activation triggers pexophagy for functional adaptation during oxidative stress under calcium deficient-conditions**

# Laxman Manandhar^1¶^, Raghbendra Kumar Dutta^1#¶^, Pradeep Devkota^1^, Arun Chhetri^1^, Xiaofan Wei^1^, Channy Park^1^, Hyug Moo Kwon^2^, and Raekil Park^1^*

^1^ Department of Biomedical Science and Engineering, Gwangju Institute of Science and Technology, Gwangju 61005, Republic of Korea

^2^ School of Life Sciences, Ulsan National Institute of Science and Technology, Ulsan, Republic of Korea

* Correspondence:

# Raekil Park, M.D., Ph.D.

# Department of Biomedical Science & Engineering,

# Gwangju Institute of Science and Technology, Gwangju 61005, Republic of Korea

# Tel.: +82-62-715-5361; Fax: +82-62-715-5309; E-mail: rkpark@gist.ac.kr

# ^¶^These authors contributed equally.

# # Current address: Department of Chemistry (Biochemistry Division) Crosley Tower, University of Cincinnati, Ohio, 45221 USA

**Figure 1.**

**
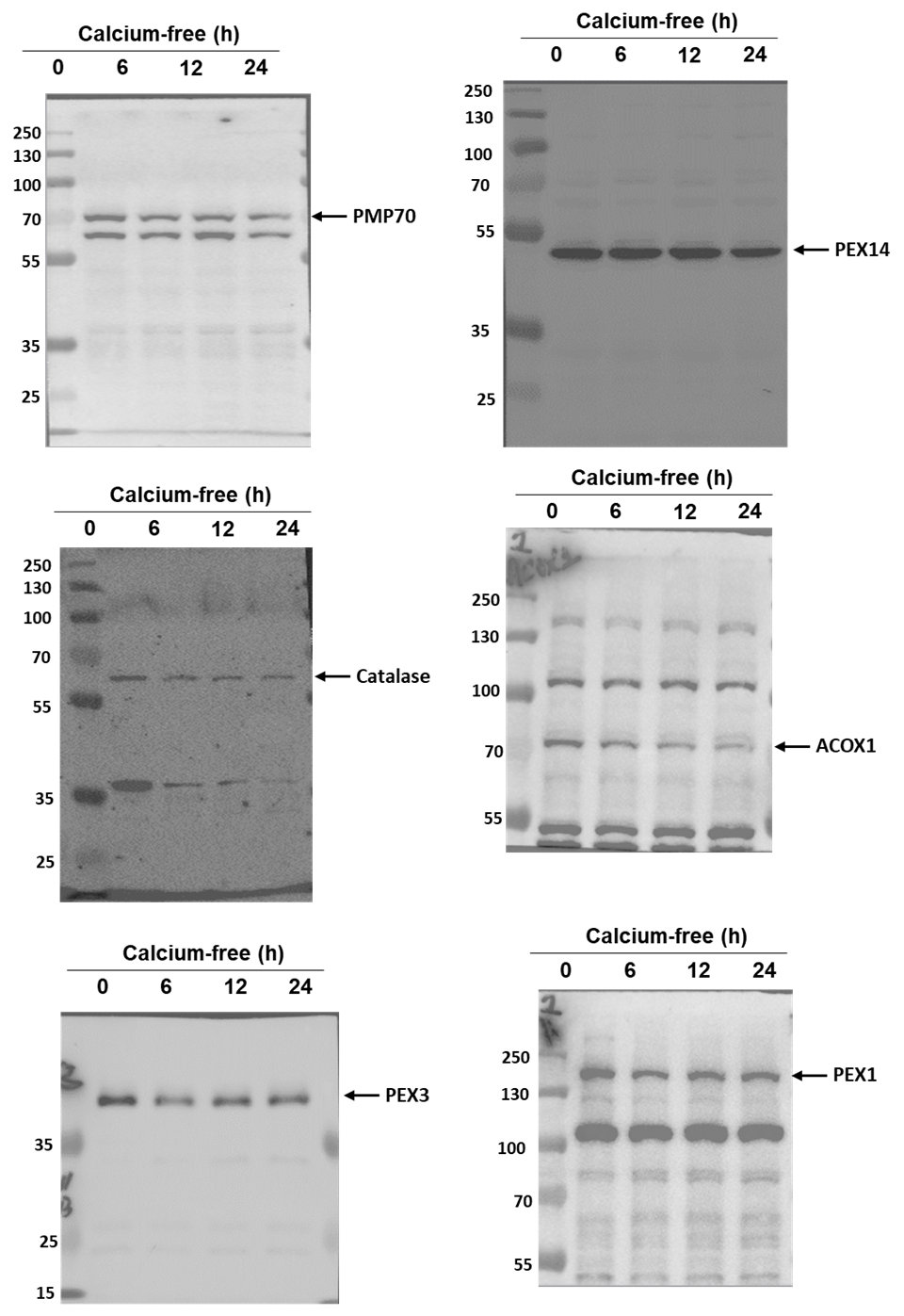
**

**
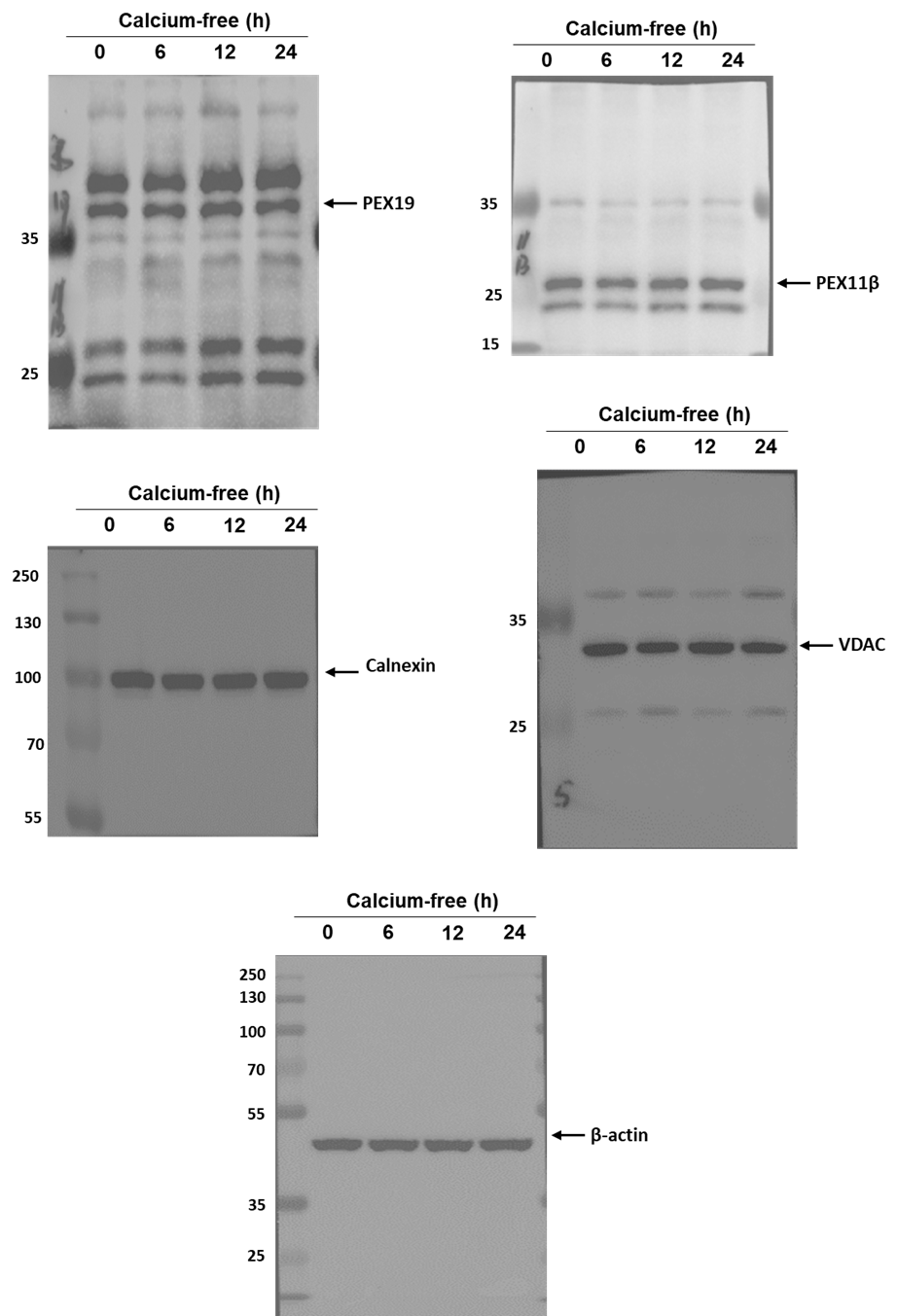
**

**Figure 3.**

**
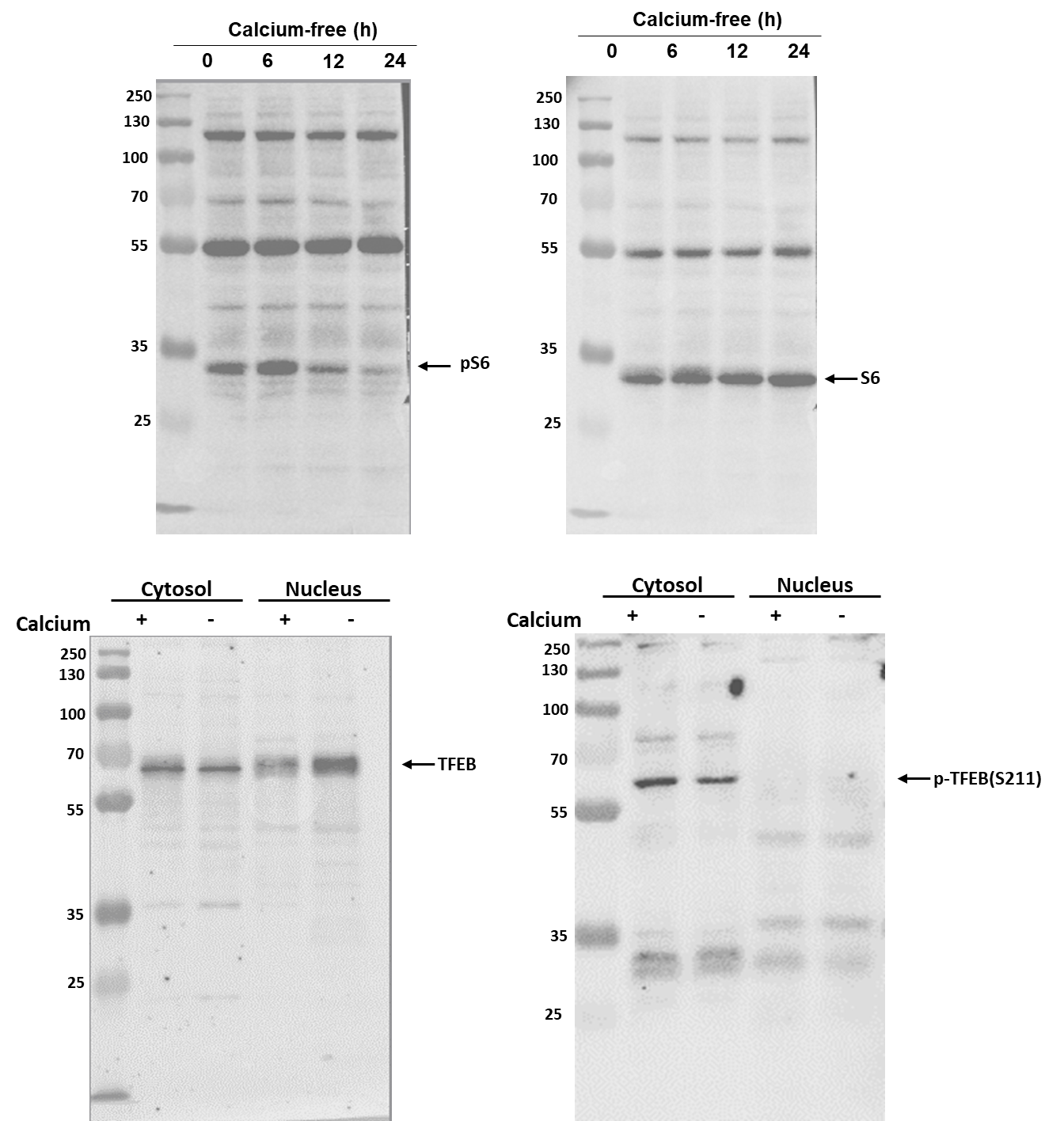
**

# **
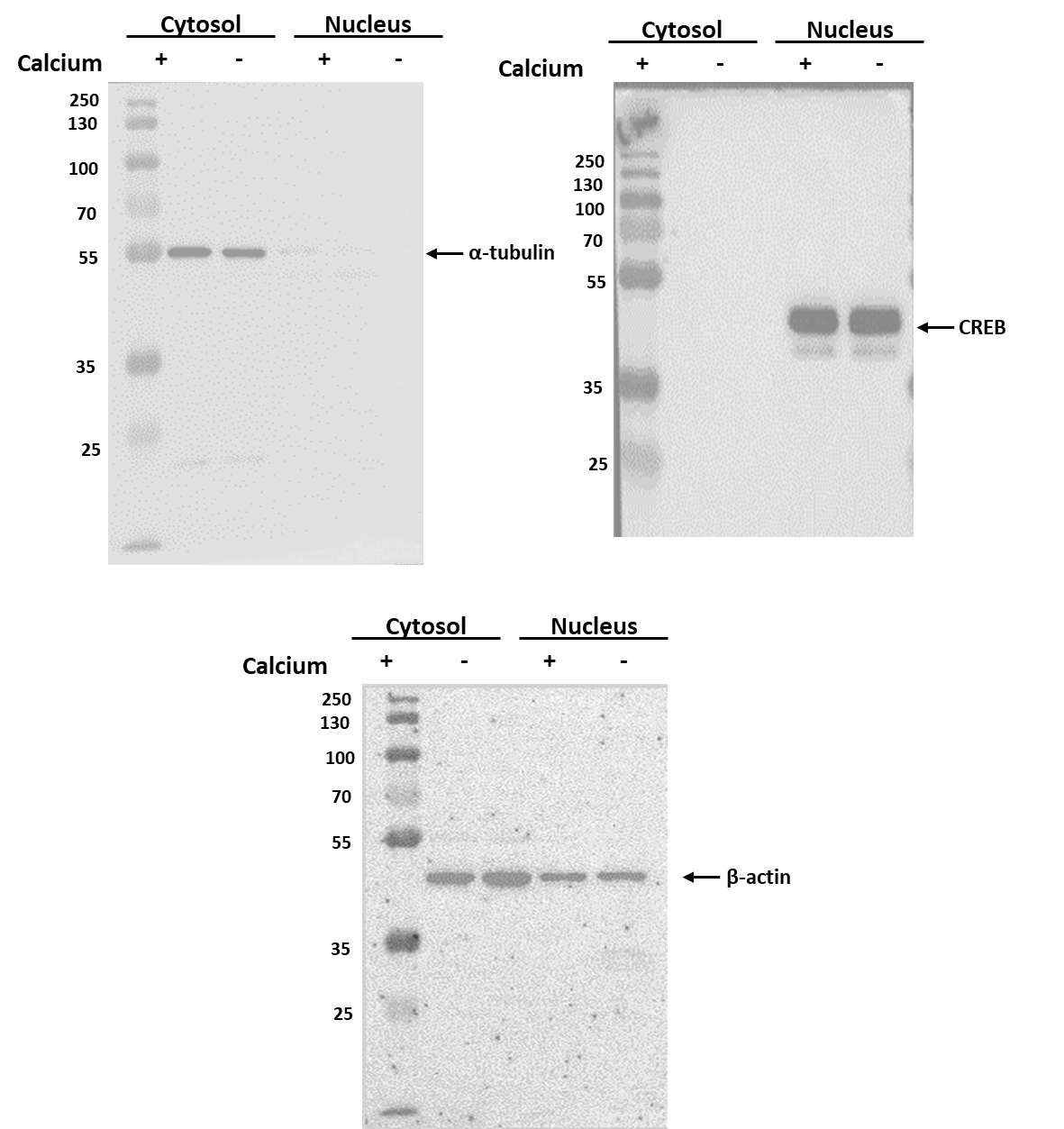
**

**Figure 4.**

**
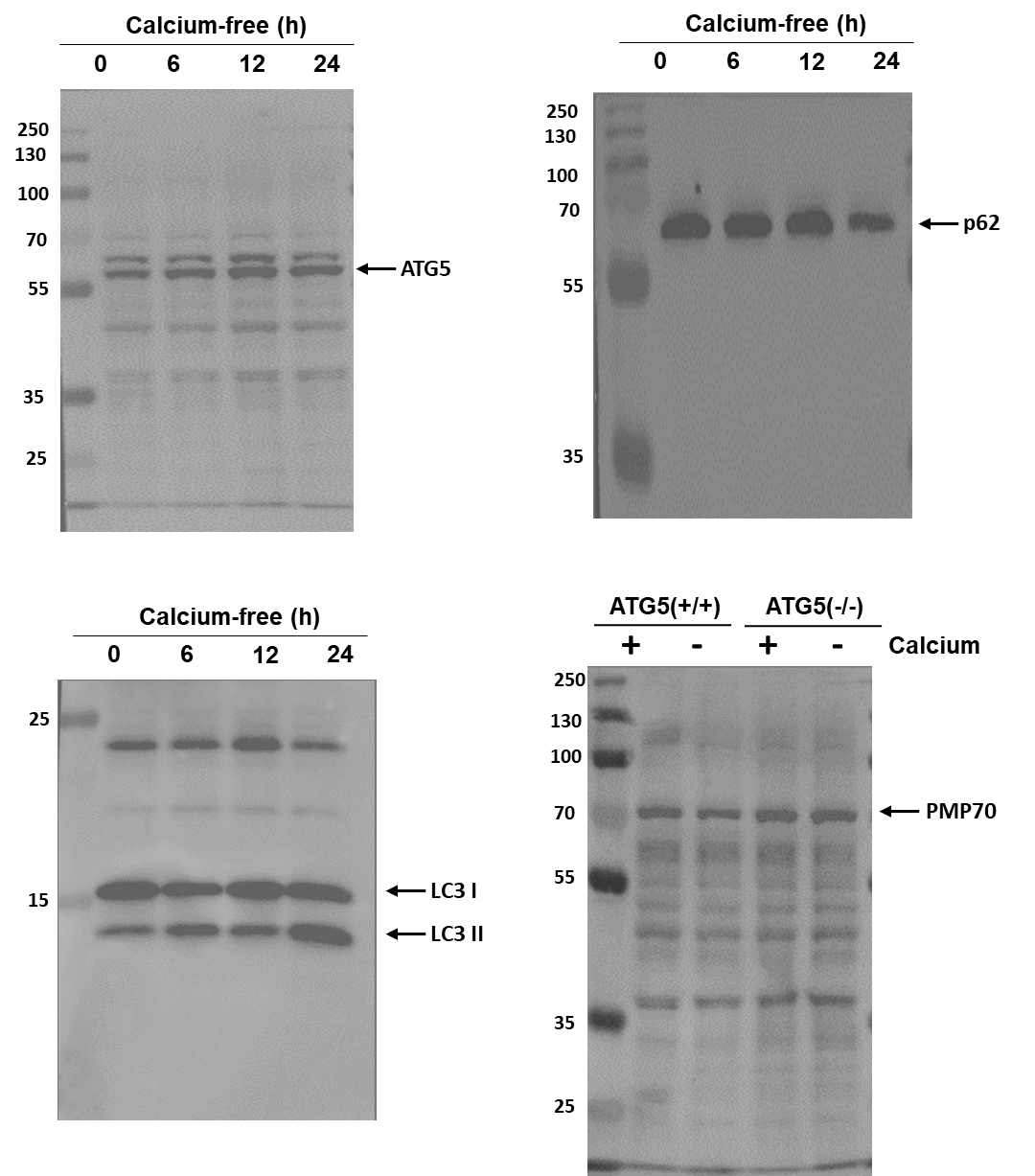
**

**
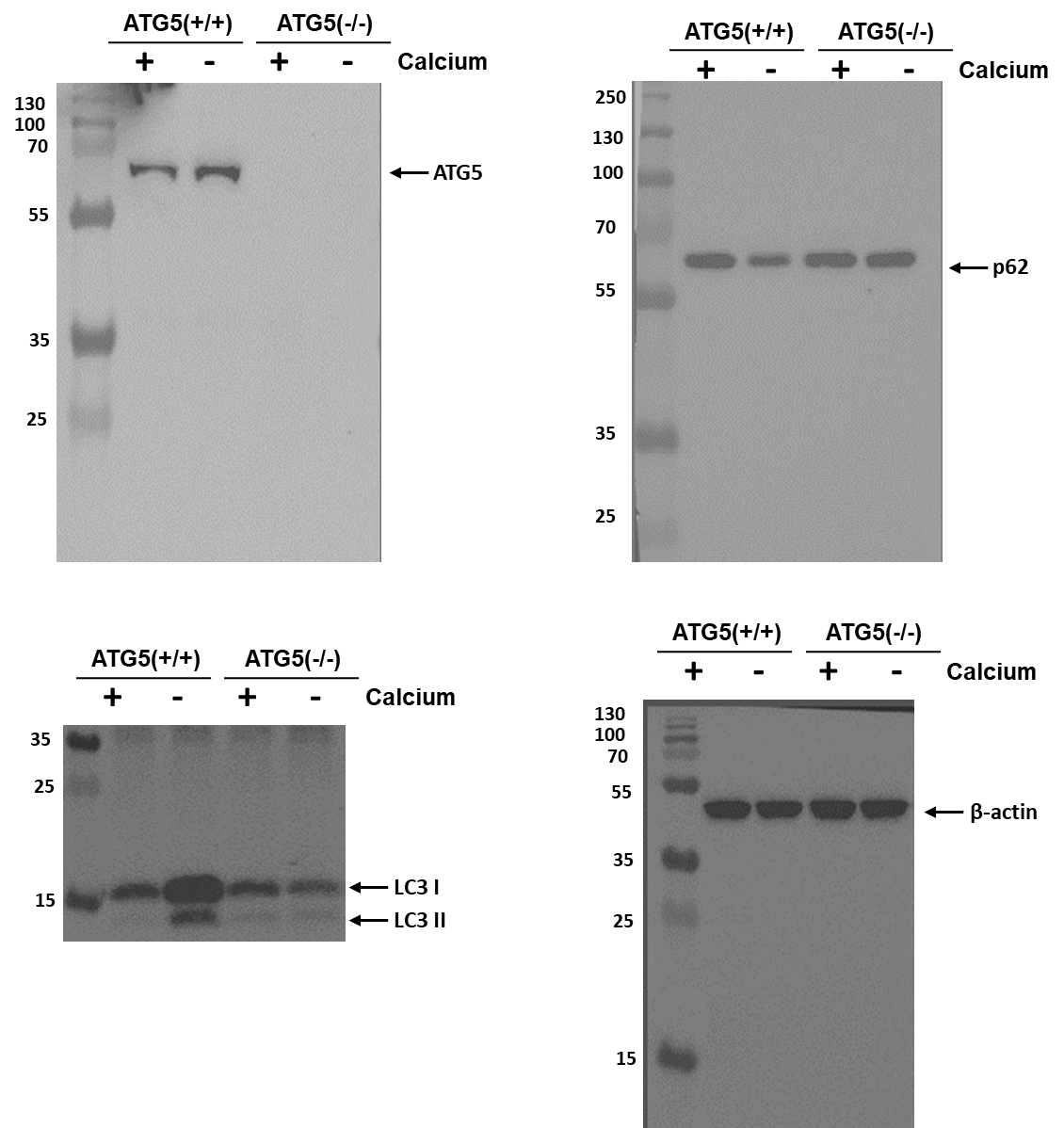
**

**Figure 5.**

**
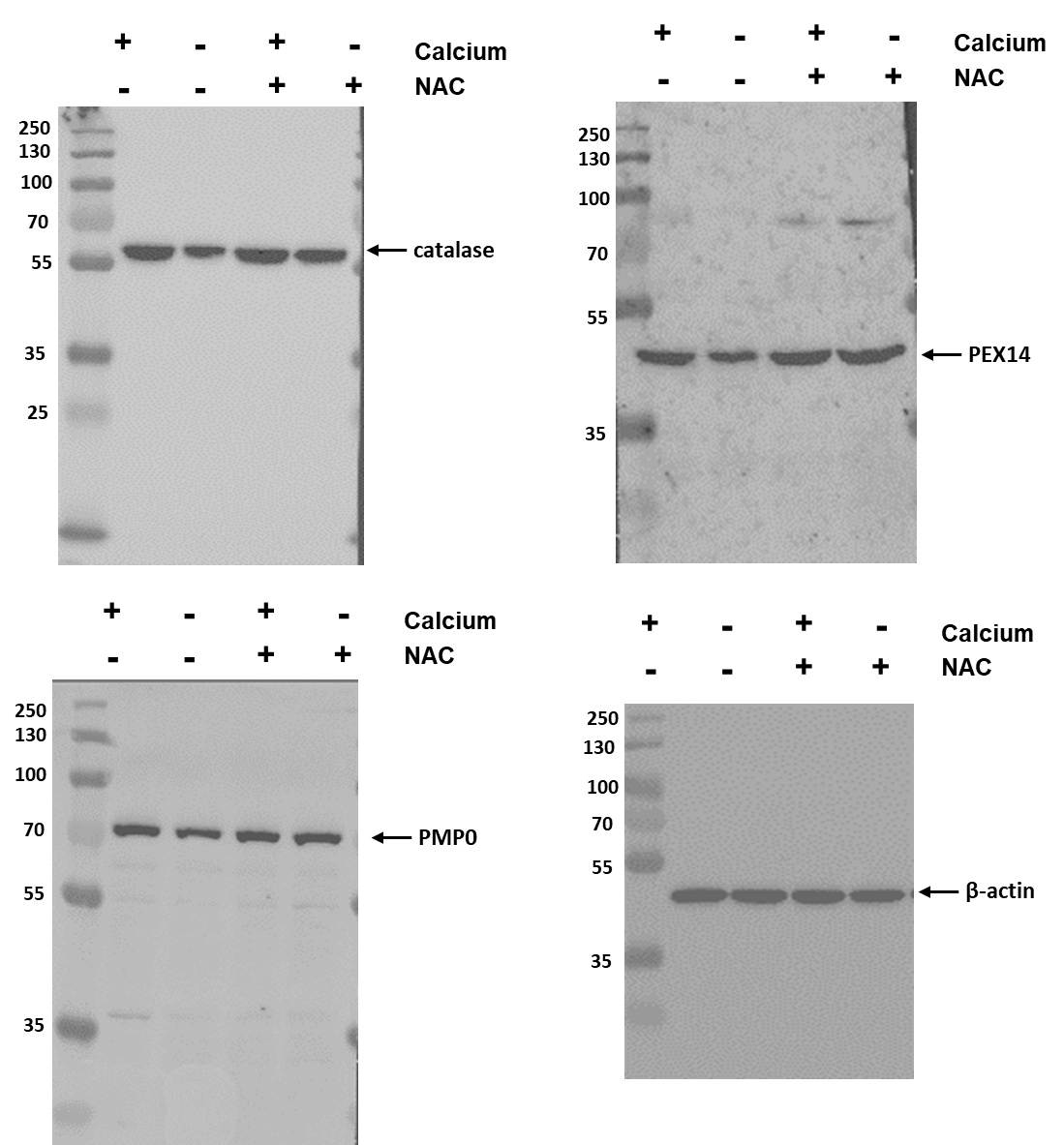
**

**
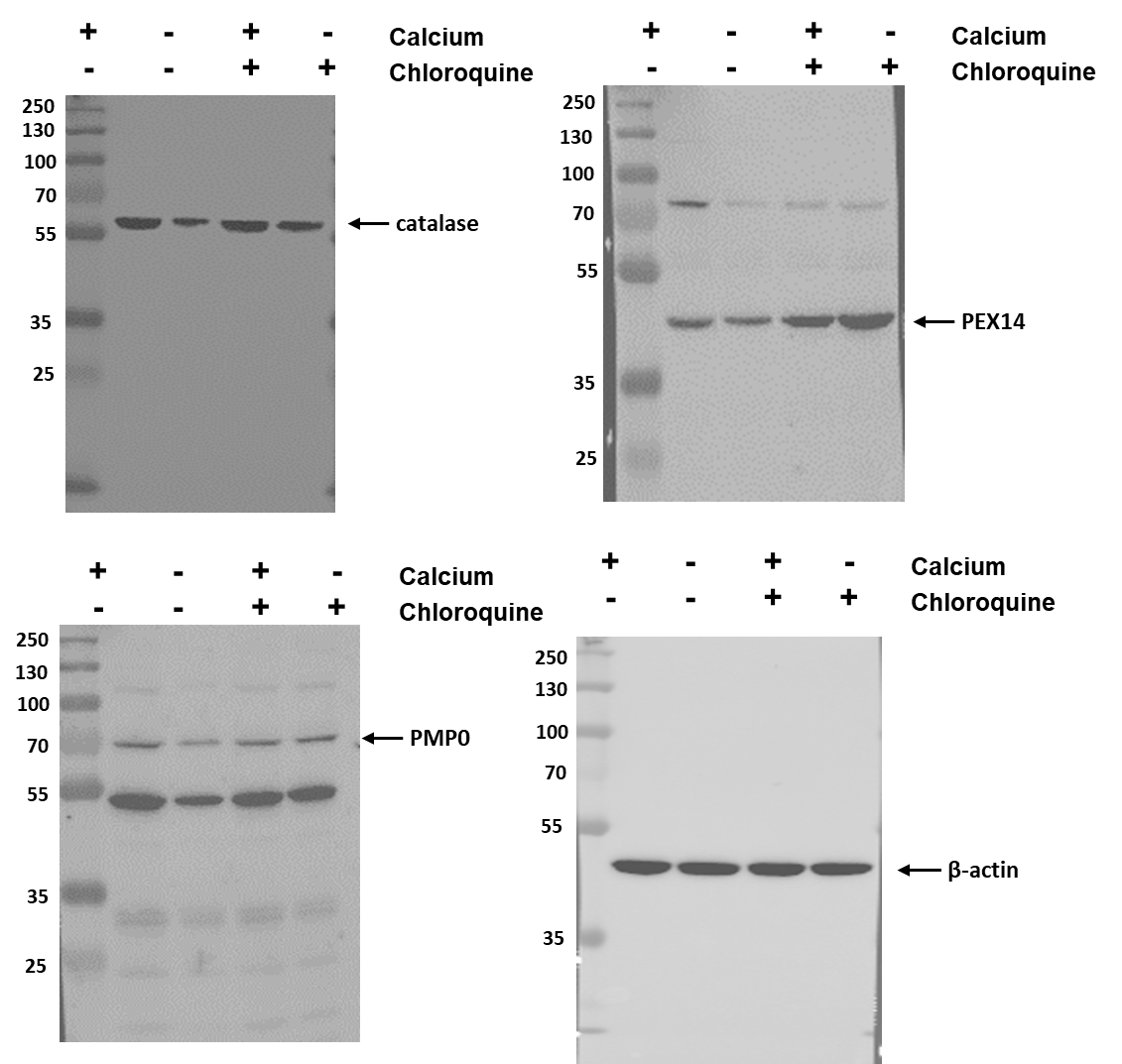
**

**
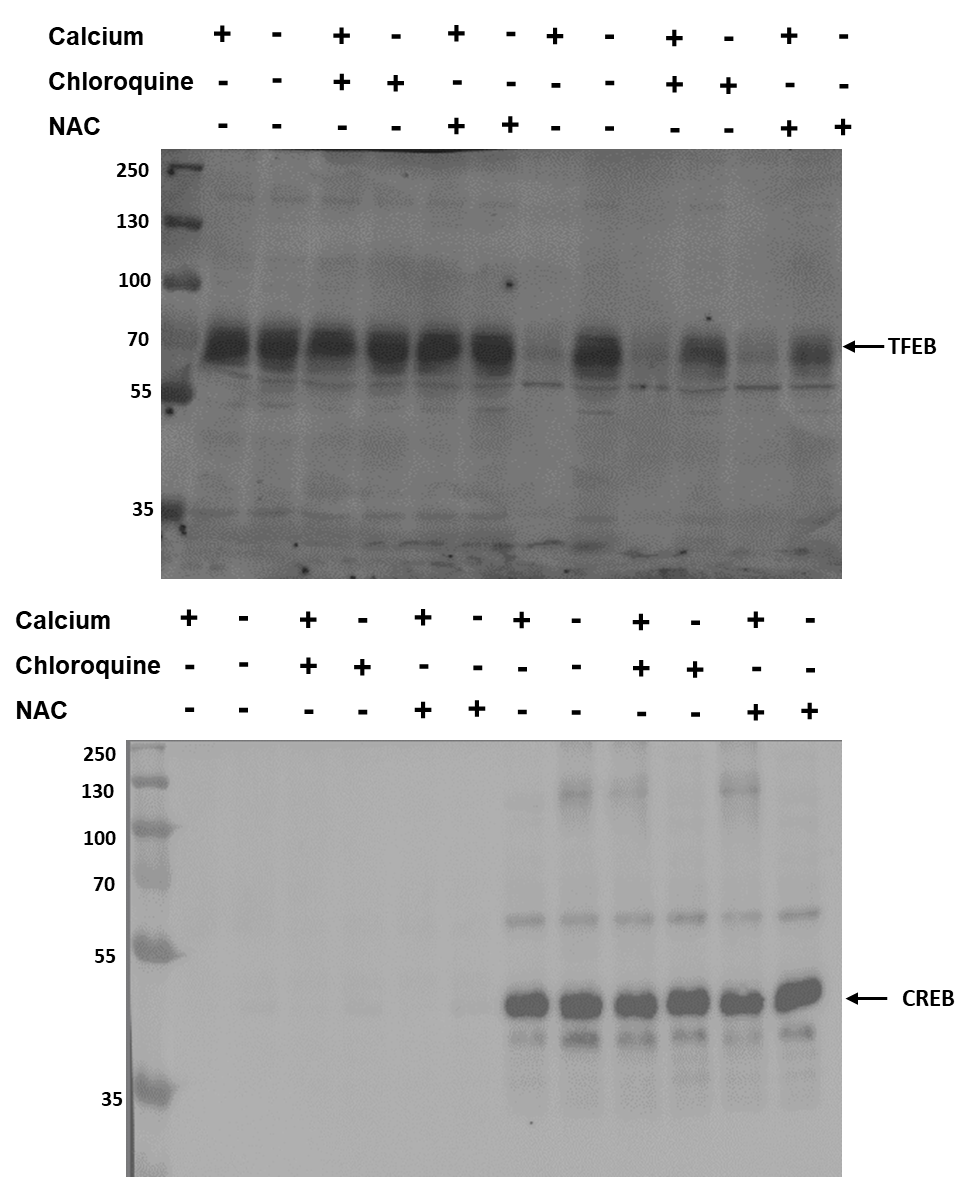
**

**
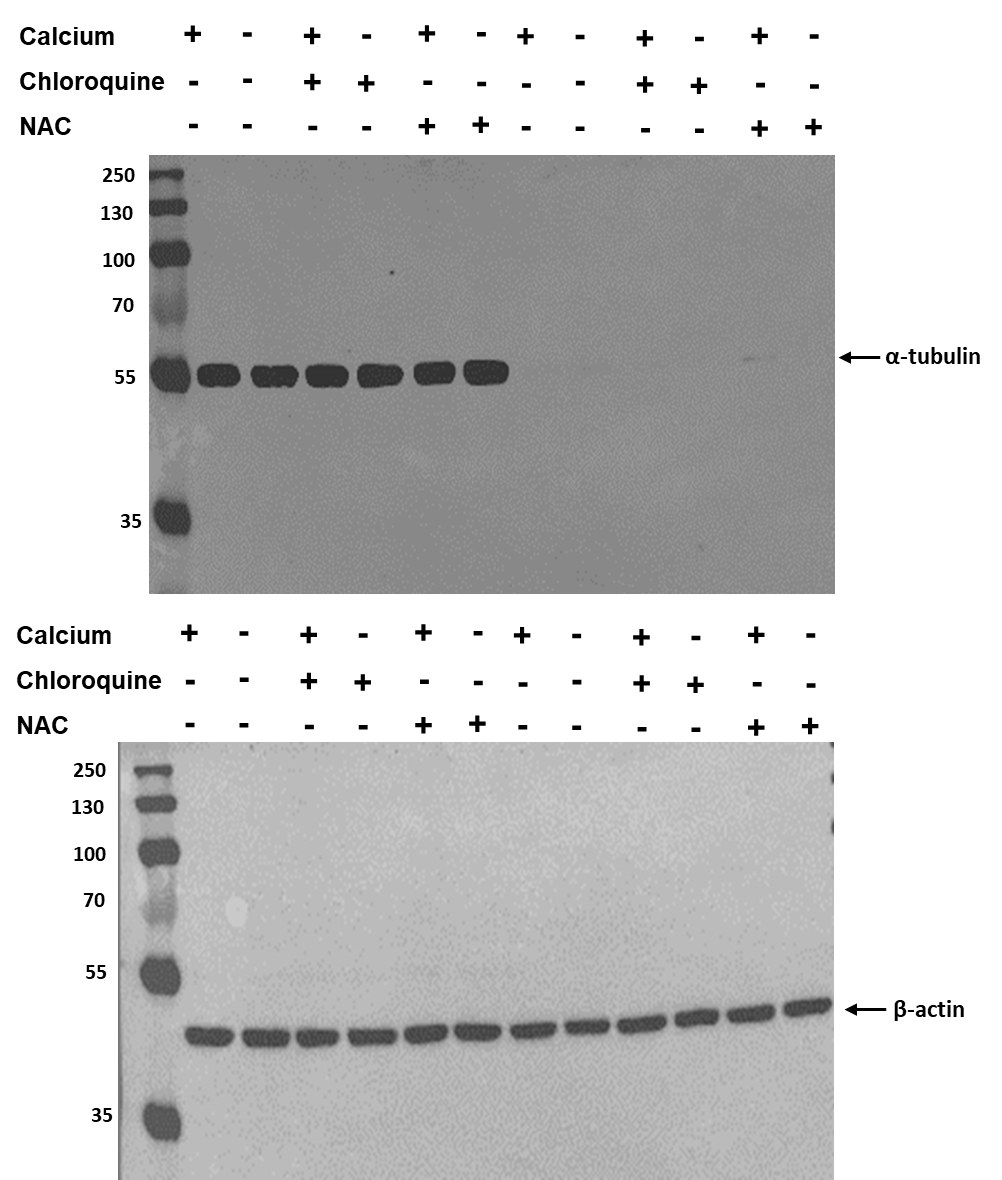
**

**Supplementary file.**

**
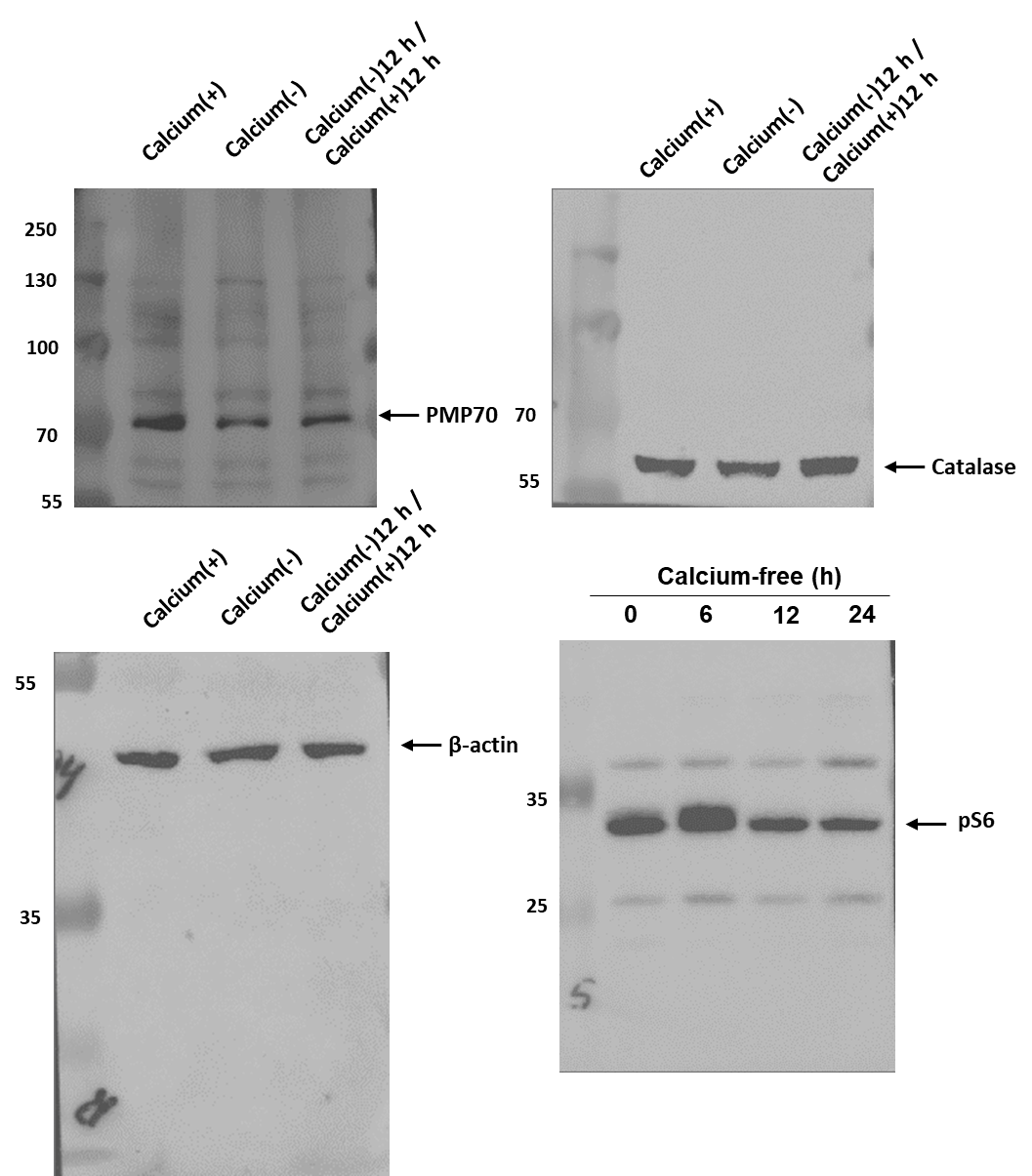
**

**
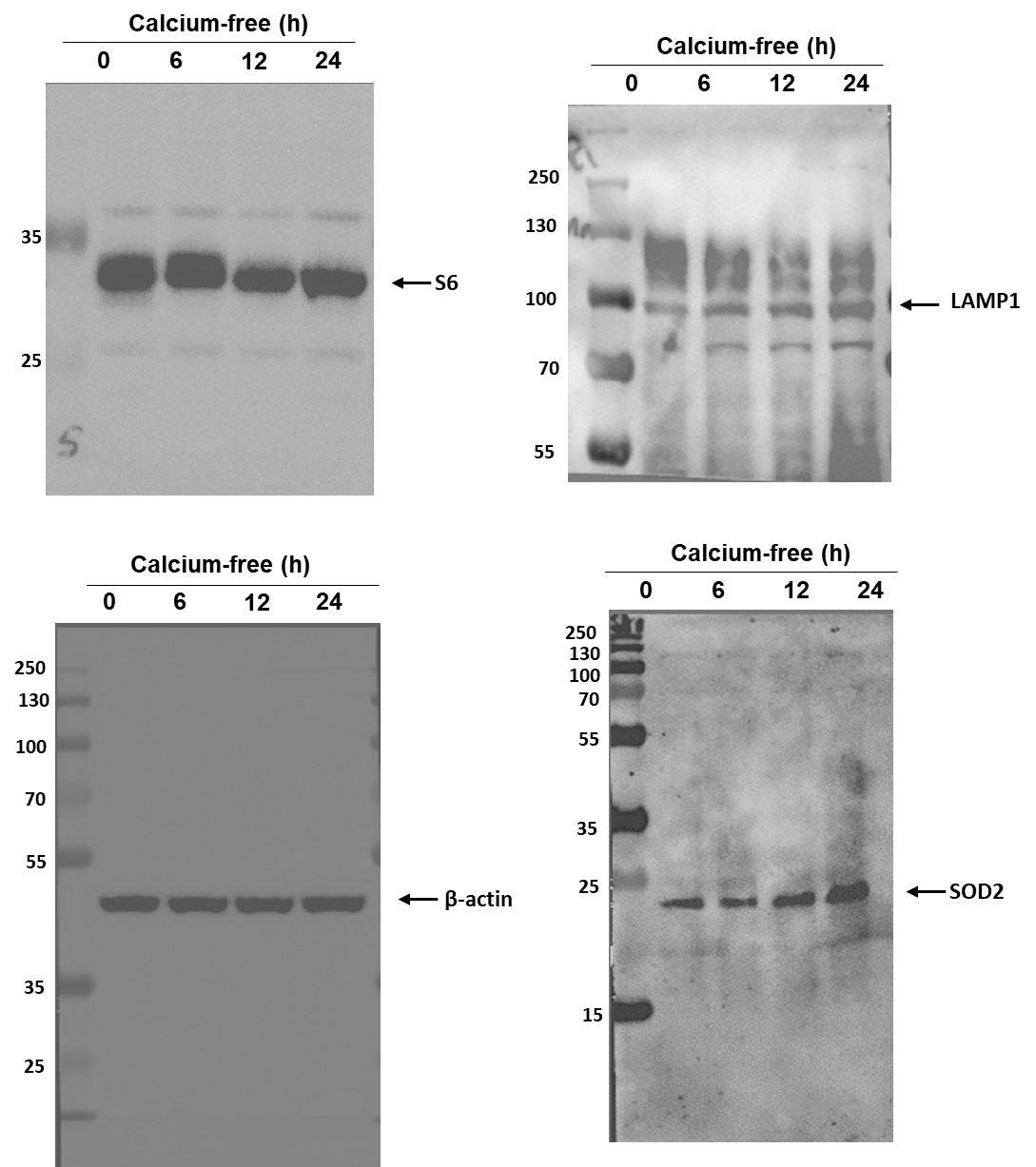
**

**
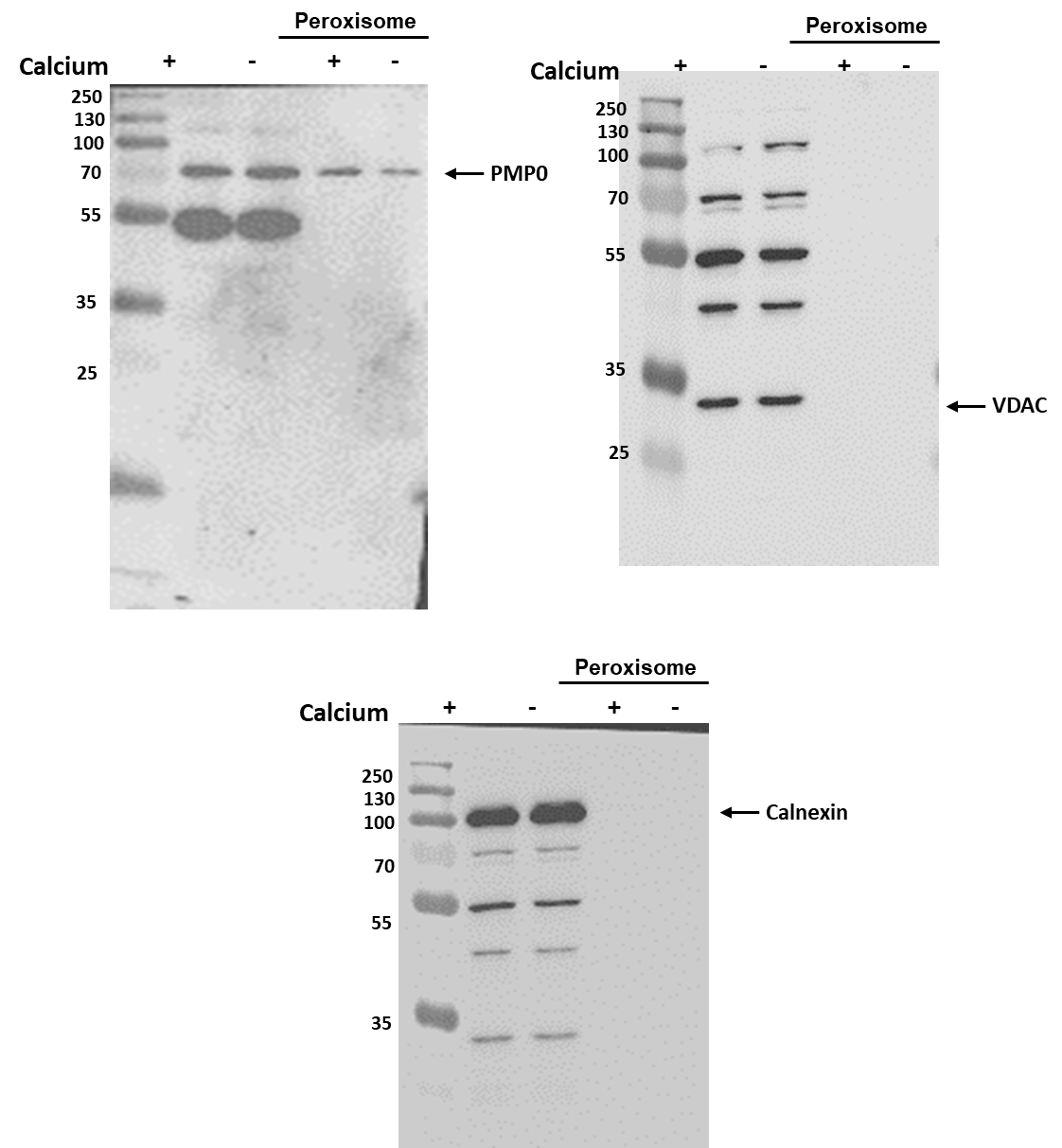
**

# **
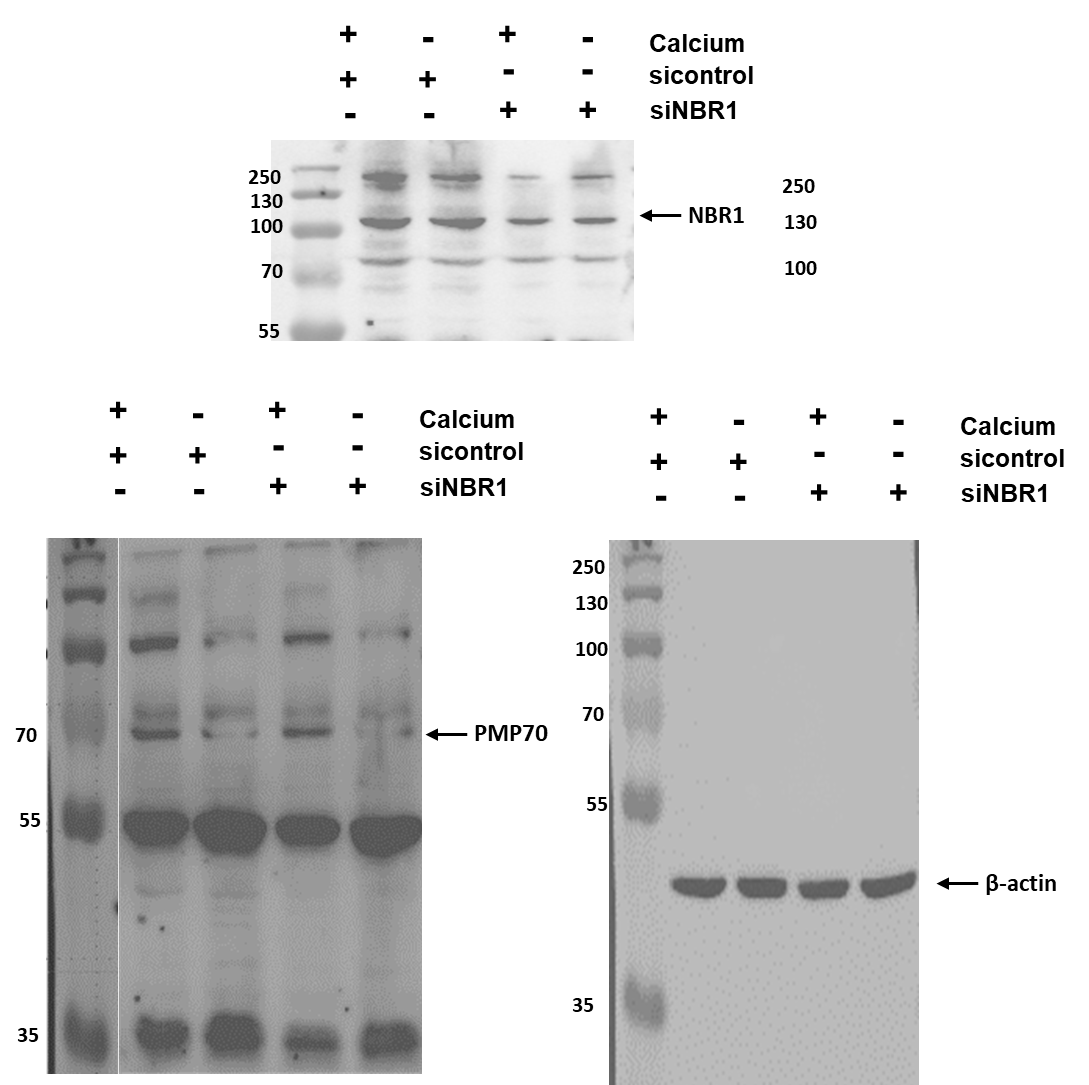
**
